# Modulating Properties of Piroxicam, Meloxicam and Oxicam Analogues against Macrophage-Associated Chemokines in Colorectal Cancer

**DOI:** 10.3390/molecules26237375

**Published:** 2021-12-05

**Authors:** Paulina Lewandowska, Izabela Szczuka, Iwona Bednarz-Misa, Berenika M. Szczęśniak-Sięga, Katarzyna Neubauer, Magdalena Mierzchała-Pasierb, Marek Zawadzki, Wojciech Witkiewicz, Małgorzata Krzystek-Korpacka

**Affiliations:** 1 Department of Biochemistry and Immunochemistry, Wroclaw Medical University, 50-368 Wroclaw, Poland; paulina.lewandowska@student.umed.wroc.pl (P.L.); izabela.szczuka@umed.wroc.pl (I.S.); iwona.bednarz-misa@umed.wroc.pl (I.B.-M.); magdalena.mierzchala-pasierb@umed.wroc.pl (M.M.-P.); 2 Department of Medicinal Chemistry, Faculty of Pharmacy, Wroclaw Medical University, 50-556 Wroclaw, Poland; berenika.szczesniak-siega@umed.wroc.pl; 3 Department and Clinics of Gastroenterology and Hepatology, Wroclaw Medical University, 50-556 Wroclaw, Poland; katarzyna.neubauer@umed.wroc.pl; 4 Department of Oncological Surgery, Regional Specialist Hospital, 51-124 Wroclaw, Poland; zawadzki@wssk.wroc.pl (M.Z.); witkiewicz@wssk.wroc.pl (W.W.); 5 Department of Physiotherapy, Wroclaw Medical University, 51-618 Wroclaw, Poland; 6 Research and Development Centre, Regional Specialist Hospital, 51-124 Wroclaw, Poland

**Keywords:** non-steroidal anti-inflammatory drugs (NSAIDs), drug repurposing, monocyte chemoattractant protein (MCP), macrophage inflammatory protein (MIP), immunotherapy, piroxicam, meloxicam, cancer-related inflammation, growth differentiation factor 15 (GDF-15), macrophage inhibitory cytokine 1 (MIC-1)

## Abstract

The mechanisms underlying the antineoplastic effects of oxicams have not been fully elucidated. We aimed to assess the effect of classic and novel oxicams on the expression/secretion of macrophage-associated chemokines (RTqPCR/Luminex xMAP) in colorectal adenocarcinoma cells, and on the expression of upstream the non-steroidal anti-inflammatory drug (NSAID)-activated genes *NAG1*, *NFKBIA*, *MYD88*, and *RELA,* as well as at the chemokine profiling in colorectal tumors. Meloxicam downregulated *CCL4* 9.9-fold, but otherwise the classic oxicams had a negligible/non-significant effect. Novel analogues with a thiazine ring substituted with arylpiperazine and benzoyl moieties significantly modulated chemokine expression to varying degree, upregulated NAG1 and *NFKBIA,* and downregulated *MYD88*. They inhibited *CCL3* and *CCL4,* and their effect on *CCL2* and *CXCL2* depended on the dose and exposure. The propylene linker between thiazine and piperazine nitrogens and one arylpiperazine fluorine substituent characterized the most effective analogue. Only *CCL19* and *CXCL2* were not upregulated in tumors, nor was *CXCL2* in tumor-adjacent tissue compared to normal mucosa. Compared to adjacent tissue, *CCL4* and *CXCL2* were upregulated, while *CCL2*, *CCL8,* and *CCL19* were downregulated in tumors. Tumor *CCL2* and *CCL7* increased along with advancing T and *CCL3,* and *CCL4* along with the N stage. The introduction of arylpiperazine and benzoyl moieties into the oxicam scaffold yields effective modulators of chemokine expression, which act by upregulating *NAG1* and interfering with NF-κB signaling.

## 1. Introduction

Colorectal cancer (CRC) is one of the most common malignancies worldwide. Its incidence is higher among individuals with a history of polyps and patients with inflammatory bowel disease [1]. Chemokines, which are involved in immune cell trafficking, constitute a link between inflammation and cancer. Moreover, they mediate interactions between tumor and stromal cells via their respective 7-transmembrane-spanning G protein-coupled receptors, facilitating cancer angiogenesis, invasion, and metastasis. As such, their signaling pathways are currently under investigation either as potential targets for immunotherapies or, on the contrary, as therapeutics (reviewed in [2]).

Monocyte chemoattractant proteins (MCPs) are members of C-C motif chemokines, and are the main chemoattractants for monocytes, but particular MCPs can also recruit other immune cells which are relevant for cancer development. Within tumors, MCP chemokines are expressed mainly by cancer cells, but their expression by tumor-associated macrophages (TAMs), cancer-associated fibroblasts (CAFs), tumor-associated neutrophils (TANs), myeloid-derived suppressor cells (MDSCs), and mesenchymal stem cells (MSCs) has been reported (reviewed in [3]). Macrophage inflammatory proteins (MIPs) are members of C-C or C-X-C motif chemokine families, acting as chemoattractants for monocytes, neutrophils, dendritic cells, and T and NK cells. In addition to cancer cells, they are expressed by TAMs, TANs, CAFs, MDSCs, and MSCs (reviewed in [4,5]). Depending on the chemokine, the type of recruited cells and the context, MCP and MIP chemokines may either enhance anti-tumor responses or confer a growth advantage for tumors [2]. In CRC, an overexpression of MCPs has been associated with a worse prognosis, while that of MIPs has been associated with a better prognosis (reviewed in [3,4]).

Non-steroidal anti-inflammatory drugs (NSAIDs) are a large group of redox-active molecules with analgesic, antipyretic, and anti-inflammatory properties, executed mainly by the inhibition of cyclooxygenase (COX) activity, and therefore the synthesis of prostanoids. Depending on NSAID, they may be selective COX-1 or COX-2 inhibitors or non-selective inhibitors of both isoforms [6]. The activity of constitutively expressed COX-1 has been shown to exert cytoprotective effects and partake in sustaining mucosal integrity. The expression of COX-2, in turn, is physiologically negligible, but is induced by stress and proinflammatory signals and growth factors [7]. The overexpression of COX-2 in the bowel, accompanied by an elevation in prostaglandin E2, has been linked with chronic inflammatory conditions [8] and cancer [7,9,10]. Consistently, NSAIDs have been shown to display promising chemopreventive and antitumor properties, especially in colorectal cancer [11,12,13,14,15,16,17].

**Figure 1:**
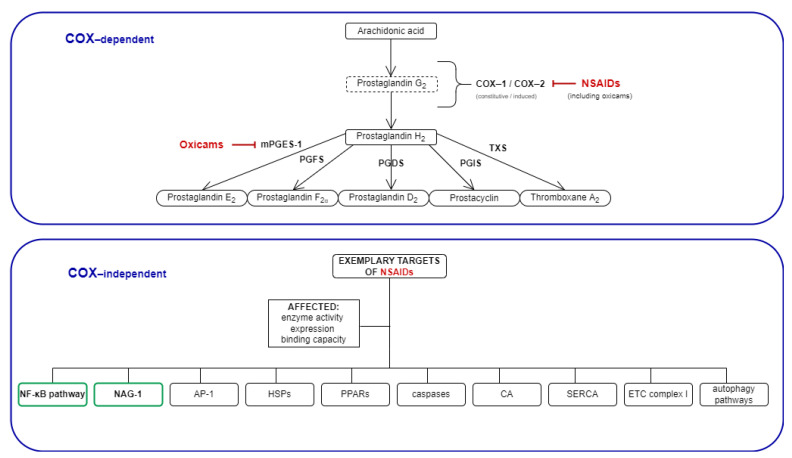
Mechanisms of NSAIDs’ action: COX-dependent and exemplary COX-independent targets relevant for cancer development. The COX-independent targets evaluated in the present study are bolded, and are indicated by a green frame. NSAIDs, non-steroidal anti-inflammatory drugs; COX, cyclooxygenase; mPGES, microsomal prostaglandin E2 synthase; PGFS, prostaglandin F synthase; PGDS, prostaglandin D synthase; PGIS, prostacyclin synthase; TXS, thromboxane synthase; NAG-1, NSAIDs-activated gene; AP-1, activator protein 1; HSPs, heat shock proteins; PPARs, peroxisome proliferator-activated receptor; CA, carbonic anhydrase; SERCA, sarco (endo)-plasmic calcium ATPase; ETC, electron transport chain.

Although they are anti-inflammatory by design and, thus, prime candidates for chemoprevention in inflammatory bowel disease patients, classic NSAIDs have been shown to exhibit substantial gastrointestinal toxicity, discouraging their application in this group of patients [18]. The gastrointestinal toxicity of NSAIDs has traditionally been attributed to their inhibitory effect on COX-1, but recently gathered data suggest a more complex mechanism [19]. Oxicams are a structurally different class of NSAIDs, distinguished by the lack of a carboxyl group. In addition, they utilize a separate binding pocket in the COX enzyme and inhibit microsomal prostaglandin E synthase-1 (mPGES-1) [20]. They downregulate redox-sensitive and inflammation-associated transcription-factors NF-κB and AP-1, and reduce their DNA-binding capacity [21]. The NSAIDs have also been demonstrated to downregulate the expression of heat shock proteins [22], known regulators of cellular-redox status [23] and chemokine chaperones (Figure 1). The oxicam protagonist, piroxicam, reduces the incidence and the size of tumors in animal models of CRC [11,12]. Noteworthily, some of the newly synthesized oxicam analogues have been shown to display reduced gastrointestinal toxicity, and are thus of interest as potential chemopreventive and/or anti-neoplastic agents [24]. Novel analogues modulate the L-arginine/nitric oxide pathway [25] and display reactive-oxygen species (ROS)-scavenging properties [26].

It is well recognized now that the antineoplastic properties of NSAIDs go beyond COX inhibition [27,28,29], and a better understanding of the underlying molecular mechanisms is crucial to enable the design of improved drugs for molecular-targeted treatment strategies in CRC [30]. The aim of the present study was to determine whether classic oxicams (piroxicam and meloxicam) and their novel analogues affect the expression and secretion of MCPs (*CCL2*/MCP-1, *CCL7*/MCP-3, and *CCL8*/MCP-2) and MIPs (*CCL3*/MIP-1α, *CCL4*/MIP-1β, *CCL19*/MIP-3β, and *CXCL2*/MIP-2) in colorectal adenocarcinoma cell lines. In order to shed some light on a mechanism of oxicam action, its impact on NAG1 (GDF15)—a growth factor belonging to the tumor growth factor (TGF)-β superfamily and an NSAID-activated gene—as well as on the expression of NFκB-associated *RELA* (encoding NFκB p65 subunit), *MYD88* (myeloid differentiation primary response 88, an adapter protein), and *NFKBIA* (encoding IκBα, an NFκB inhibitor) was additionally evaluated. In the light of the paucity of clinical data on MCP and MIP in CRC other than prototypical MCP-1 and MIP-1, their transcriptional patterns in transformed and non-transformed colorectal mucosa from CRC patients in reference to normal colonic tissue, cancer pathology, and the concentration of circulating chemokines were investigated.

## 2. Results

### 2.1. MCP and MIP Expression in Colonic Adenocarcinoma Cell Lines

**Figure 2:**
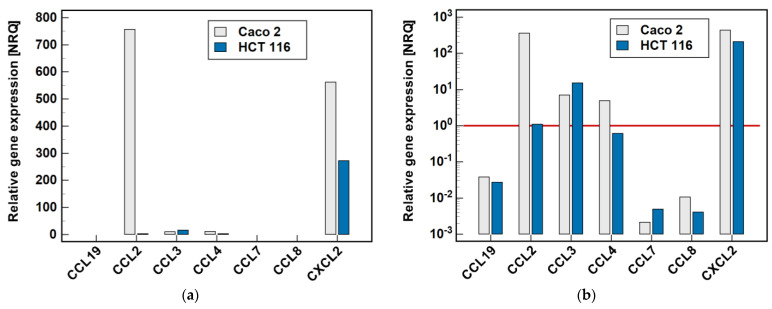
Relative expression of MCPs and MIPs in the Caco-2 and HCT 116 colonic adenocarcinoma cell lines: (**a**) non-logarithmized data; (**b**) data presented on a log-scale. The data are presented as normalized relative quantities (NRQ), normalized against *GAPDH* expression and expressed in relation to the geometric mean of global expression of all of the genes across both cell lines. The gene expression was analyzed in the control (untreated) cells, *n* = 9 per line. The red horizontal line represents the global mean expression.

Two cell lines, namely Caco-2 and HCT 116, were tested by RTqPCR for the expression level of MCP and MIP chemokines. As depicted in Figure 2, there was great variability in the expression level of particular genes. In the Caco-2 cell line, *CCL2* and *CXCL2* were highly expressed. The expression level of *CCL3* and *CCL4*, although markedly lower, still allowed for their reliable quantification. In the HCT 116 cell line, *CXCL2* expression was the highest, although lower than in Caco-2, and that of *CCL3* could still be credibly measured. The expression of the remaining genes was too low to be reliably quantified, and therefore they were not analyzed further.

### 2.2. Effect of Oxicams on MCP and MIP Expression in Colonic Adenocarcinoma Cell Lines

The Caco-2 and HCT 116 cells were treated with oxicams: the classic drugs piroxicam (compound #**6**) and meloxicam (compound #**7**), and their five new analogues, denoted as compounds #**1**–**5**. In order to analyze the effect of time, the cells were incubated with 200 μM drugs for 6 and 24 h. In order to analyze the dose–response, the cells were incubated for 24 h with 5, 50 and 200 μM concentrations of oxicams.

#### 2.2.1. Time-Dependent Response to Oxicams

**Figure 3:**
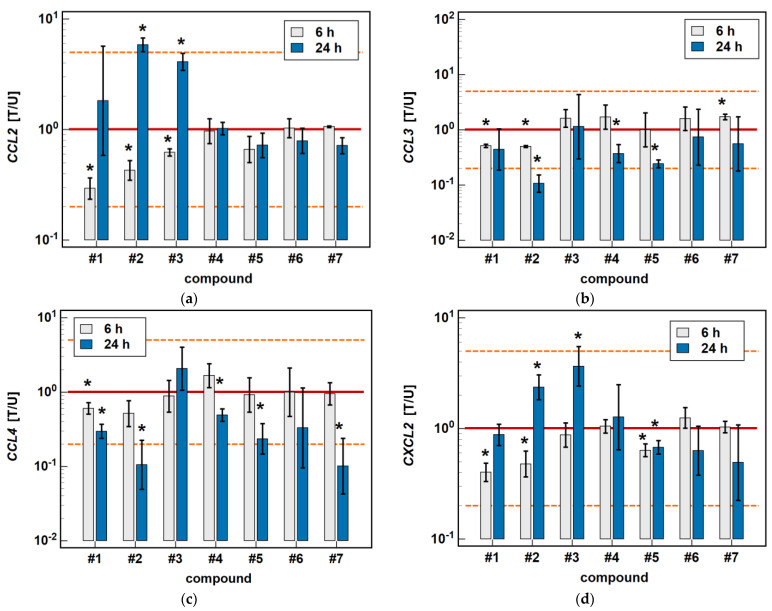
Time-dependent effect of oxicams on MCP and MIP expression in Caco-2 cells: (**a**) *CCL2*; (**b**) *CCL3*; (**c**) *CCL4*; (**d**) *CXCL2*. The bars and whiskers represent the mean ± SD expression ratio of the normalized relative quantities [NRQ] obtained for cells treated with 200 μM oxicams for 6 and 24 h, and the corresponding untreated controls [T/U]. The significant differences between the treated and untreated cells, analyzed using a *t*-test for paired samples, are indicated by asterisks (*). The expression ratio of 1 (no change) is indicated by a red solid reference line, while the expression ratio of 5 (5-fold upregulation) and the expression ratio of 0.2 (5-fold downregulation) are indicated by an orange dashed reference line. Compound #**6** denotes piroxicam, and #**7** denotes meloxicam.

In Caco-2 cells, the classic oxicams tended to downregulate *CCL2*, *CCL3*, *CCL4* and *CXCL2* expression after 24-h treatment (1.3 to 3.0-fold), but the effect was significant only in the case of meloxicam and *CCL4* expression (9.9-fold downregulation). A shorter incubation resulted in the slight upregulation of *CCL3* and *CXCL2* (by 1.3 to 1.6-fold), which was significant in the case of meloxicam and *CCL3* (1.7-fold upregulation) (Figure 3).

Compounds #**1** and #**2** downregulated *CCL3* and *CCL4* expression in a time-dependent manner. *CCL3* was downregulated by #**1** 2- and 2.4-fold, and by #**2** 2- and 9.4-fold at 6 and 24 h, respectively. *CCL4* was downregulated by #**1** 1.7- and 3.4-fold, and by #**2** 1.9- and 9.5-fold at 6 and 24 h, respectively (Figure 3).

Compounds #**4** and #**5** downregulated *CCL3* (2.7- and 4.1-fold, respectively) and *CCL4* (2- and 4.3-fold, respectively) only in the 24-h cultures, while the shorter treatment had no effect on gene expression (Figure 3).

The expression of *CCL2* and *CXCL2* was affected primarily by compounds #**1** to **3**, and the effect was either inhibitory or stimulatory depending on the length of exposure. A shorter incubation with #**1** to **3** downregulated *CCL2* (3.4-, 2.3- and 1.6-fold, respectively) and with #**1** and #**2** downregulated *CXCL2* (2.5- and 2.1-fold, respectively). In turn, longer incubation with #**2** and #**3** upregulated *CCL2* (5.8- and 4.1-fold, respectively) and *CXCL2* (2.4- and 3.7-fold, respectively). Compound #**5** had a slightly inhibitory effect on *CXCL2* expression as well, although without a difference between the 6- and 24-h treatment (1.6- and 1.5-fold, respectively) (Figure 3).

**Figure 4:**
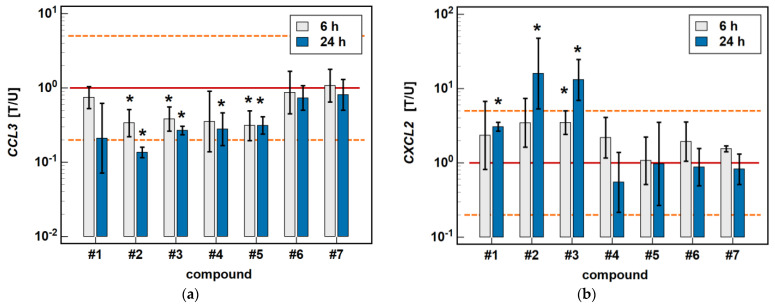
Time-dependent effect of oxicams on MCP and MIP expression in HCT 116 cells: (**a**) *CCL3*; (**b**) *CXCL2*. The bars and whiskers represent the mean ± SD expression ratio of the normalized relative quantities [NRQ] obtained for cells treated with 200 μM oxicams for 6 and 24 h, and the corresponding untreated controls [T/U]. The significant differences between the treated and untreated cells, analyzed using a *t*-test for paired samples, are indicated by asterisks (*). An expression ratio of 1 (no change) is indicated by a red solid reference line, while an expression ratio of 5 (5-fold upregulation) and an expression ratio of 0.2 (5-fold downregulation) are indicated by an orange dashed reference line. Compound #**6** denotes piroxicam, and #**7** denotes meloxicam.

In the HCT 116 cell line, *CCL3* was downregulated by compound #**2** (3- and 7.4-fold after 6 and 24-h treatment) and compound #**3** (2.6- and 3.7-fold, respectively) in a time-dependent manner. Compound #**5** downregulated *CCL3* to the same degree (3.2-fold) independently from the length of treatment, and the inhibitory effect of compound #**4** (3.6-fold) was significant only in the 24-h cultures (Figure 4).

The expression of *CXCL2* was upregulated by compounds #**1** (2.3-fold at 6 h, and 3.1-fold at 24 h), compound #**2** (3.5- and 16-fold, respectively), and compound #**3** (3.5- and 13.1-fold, respectively) in a time-dependent manner. The stimulatory effect in the 6-h cultures did not reach statistical significance in the case of compounds #**1** and #**2** (Figure 4).

#### 2.2.2. Dose-Dependent Response to Oxicams

**Figure 5:**
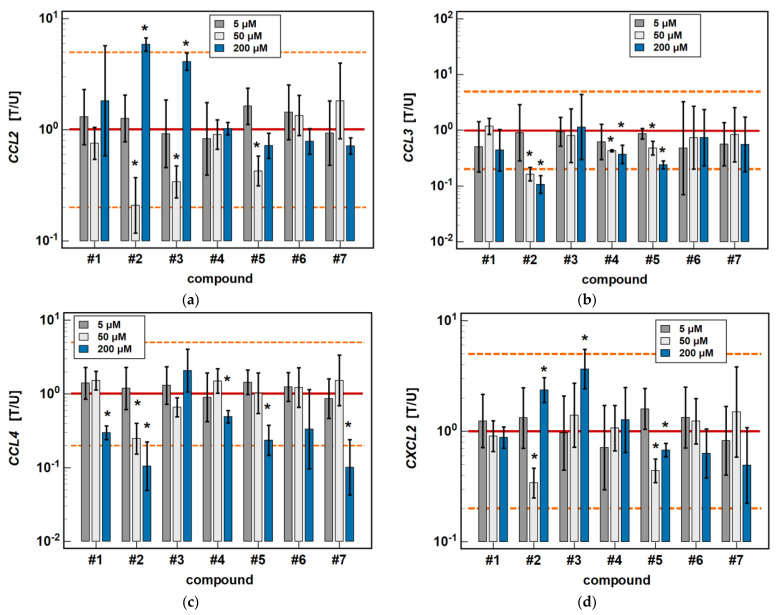
Dose-dependent effect of oxicams on MCP and MIP expression in Caco-2 cells: (**a**) *CCL2*; (**b**) *CCL3*; (**c**) *CCL4*; (**d**) *CXCL2*. The bars and whiskers represent the mean ± SD expression ratio of the normalized relative quantities [NRQ] obtained for cells treated with 5, 50, and 200 μM oxicams for 24 h, and the corresponding untreated controls [T/U]. The significant differences between the treated and untreated cells, analyzed using a *t*-test for paired samples, are indicated by asterisks (*). An expression ratio of 1 (no change) is indicated by a red solid reference line, while an expression ratio of 5 (5-fold upregulation) and an expression ratio of 0.2 (5-fold downregulation) are indicated by an orange dashed reference line. Compound #**6** denotes piroxicam, and #**7** denotes meloxicam.

In Caco-2 cells, compounds #**2**, #**4** and #**5** dose-dependently downregulated *CCL3* and *CCL4* expression. The inhibitory effect of the 50 μM concentration was significant for all of these compounds in the case of *CCL3* expression (6.2-, 2.3-, and 2.1-fold, respectively), and only for #**2** (4-fold) in the case of *CCL4* expression (Figure 5).

As for the 6-h cultures treated with 200 μM, a lower dose (50 μM) of compounds #**2** (4.8-fold) and #**3** (2.9-fold) inhibited *CCL2* expression, and compound #**2** inhibited *CXCL2* (2.9-fold), while treatment with 200 μM concentrations had the opposite effect (Figure 5).

**Figure 6:**
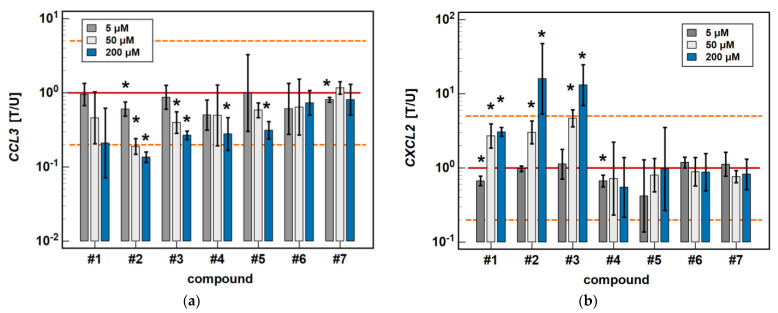
Dose-dependent effect of oxicams on MCP and MIP expression in HCT 116 cells: (**a**) *CCL3*; (**b**) *CXCL2*. The bars and whiskers represent the mean ± SD expression ratio of the normalized relative quantities [NRQ] obtained for cells treated with 5, 50, and 200 μM oxicams for 24 h, and the corresponding untreated controls (treated-to-untreated [T/U] ratio). The significant differences between the expression in treated and untreated cells, analyzed using a *t*-test for paired samples, are indicated by asterisks (*). No fold-change (T/U = 1) is indicated by a red solid reference line, and five-fold upregulation (T/U = 5) or downregulation (T/U = 0.2) is indicated by an orange dashed reference line. Compound #**6** denotes piroxicam, and #**7** denotes meloxicam.

In HCT 116 cells, compounds #**2**, #**3**, and #**5** dose-dependently downregulated *CCL3* expression. In addition to 200 μM, the effects of both a 5 and 50 μM dose were significantly inhibitory (1.7- and 5.3-fold, respectively) for compound #**2**. In the case of compound #**3**, the effect was significant for a 50 μM dose (2.5-fold), and there was a clear tendency for compound #**5** (downregulation 1.7-fold, *p* = 0.055). The 5 μM dose of meloxicam downregulated *CCL3* 1.3-fold (Figure 6).

Compounds #**1** to **3** had a stimulatory effect on *CXCL2* expression. The 5 μM dose was slightly inhibitory in the case of compound #**1** (1.5-fold), and had no impact in the case of compounds #**2** and #**3**. In turn, the 50 μM dose was significantly stimulatory for all of these compounds (2.7-, 3-, and 4.7-fold, respectively) (Figure 6).

### 2.3. Effect of Oxicams on MCP and MIP Secretion by Caco-2 Cells

The oxicam effect on MCP and MIP chemokines at the protein level was tested on Caco-2 cells. The concentrations of MCP-1/CCL2, MIP-1α/CCL3, MIP-1β/CCL4 and MCP-3/CCL7 released into the medium were simultaneously determined using Luminex xMAP technology.

**Figure 7:**
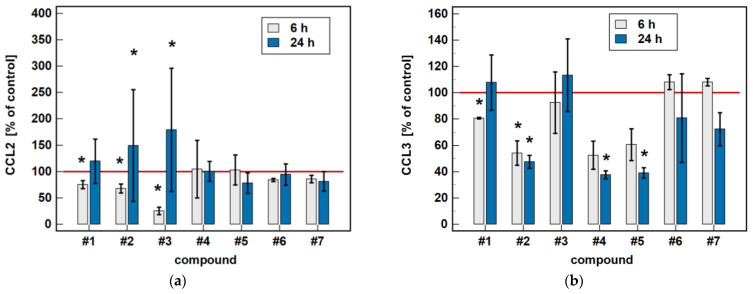

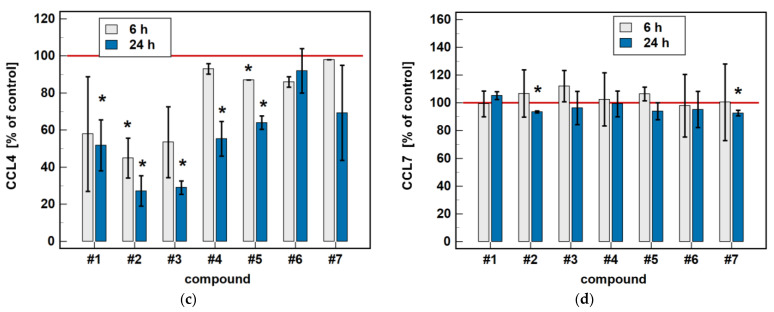
Time-dependent effect of oxicams on MCPs and MIPs secretion in Caco-2 cells: (**a**) MCP-1/CCL2; (**b**) MIP-1α/CCL3; (**c**) MIP-1β/CCL4; (**d**) MCP-3/CCL7. The bars represent the mean ± SD (whiskers) ratio of the chemokine concentration in the medium from cells treated with 200 μM oxicams for 6 and 24 h, as compared to untreated controls, expressed as a percentage. The significant differences between the secretion from treated and untreated cells, analyzed using a *t*-test for one mean, are indicated by asterisks (*). The reference secretion of the control cells is indicated by a red solid line. Compound #**6** denotes piroxicam, and #**7** denotes meloxicam.

Compared to untreated cells, compounds #**1** to **5** downregulated MIP-1β/CCL4 secretion. Likewise, compounds #**1**, #**2**, #**4** and #**5** downregulated the secretion of MIP-1α/CCL3. Except for MIP-1α/CCL3 and compound #**1**, the effect was more marked in the 24-h cultures. Compound #**2** and meloxicam (#**7**) also had a slight inhibitory effect in 24-h cultures on MCP-3/CCL7 secretion (Figure 7).

The oxicam effect on the secretion of MCP-1/CCL2 mimicked that of *CCL2* expression. A shorter treatment with #**1**, #**2**, and #**3** inhibited MCP-1/CCL2 secretion, while a longer incubation increased the MCP-1/CCL2 release (Figure 7).

**Figure 8:**
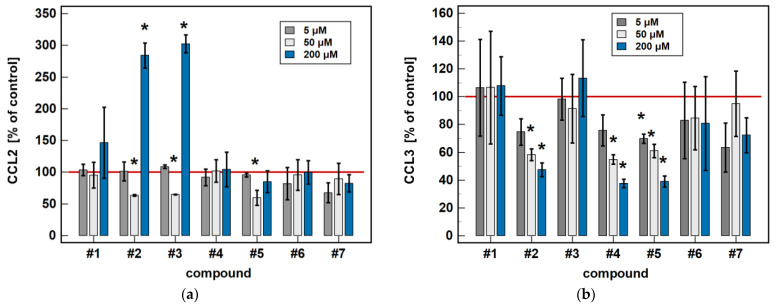

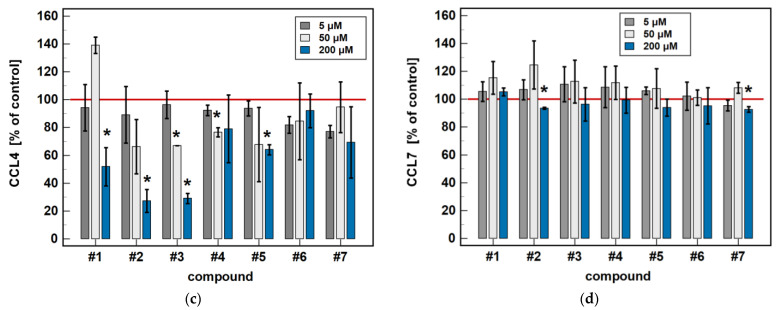
Dose-dependent effect of oxicams’ MCP and MIP secretion in Caco-2 cells: (**a**) MCP-1/CCL2; (**b**) MIP-1α/CCL3; (**c**) MIP-1β/CCL4; (**d**) MCP-3/CCL7. The bars represent the mean ± SD (whiskers) ratio of the chemokine concentration in the medium from cells treated with 5, 50 and 200 μM oxicams for 24 h, as compared to untreated controls, expressed as a percentage. The significant differences between the secretion from treated and untreated cells, analyzed using a *t*-test for one mean, are indicated by asterisks (*). The reference secretion of the control cells is indicated by a red solid line. Compound #**6** denotes piroxicam, and #**7** denotes meloxicam.

In a dose-dependence experiment, an increasing concentration of compounds #**2**, #**4**, and #**5** decreased the MIP-1α/CCL3 secretion in a stepwise manner. Likewise, the MIP-1β/CCL4 secretion decreased along with the increasing concentration of compounds #**1** to **5** (Figure 8).

Regarding MCP-1/CCL2 secretion, 5 μM concentrations of compounds #**2**, #**3**, and #**5** had no effect, while a 50 μM concentration was significantly inhibitory and, in the case of compounds #**2** and #**3**, a 200 μM dose was significantly stimulatory (Figure 8).

### 2.4. Effect of Oxicams on NAG1 Expression in Caco-2 and HCT 116 Cells

In order to explore the COX-independent mechanism of oxicam action, the potential effect of classic oxicams and their novel analogues on the expression of NSAID-activated gene-1 (*NAG1*) was determined.

**Figure 9:**
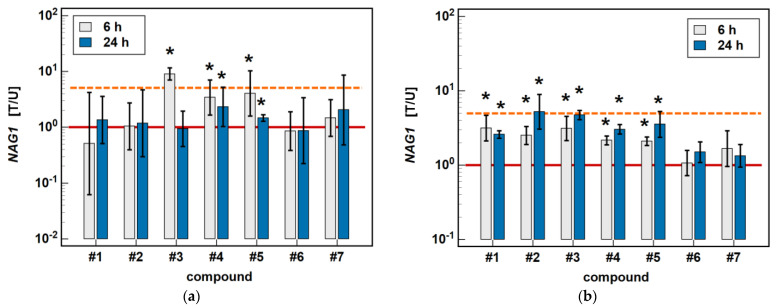
Time-dependent effect of oxicams on *NAG1* expression in (**a**) Caco-2 cells and (**b**) HCT 116 cells. The bars and whiskers represent the mean ± SD expression ratio of the normalized relative quantities [NRQ] obtained for cells treated with 200 μM oxicams for 6 and 24 h, and the corresponding untreated controls [T/U]. The significant differences between the treated and untreated cells, analyzed using a *t*-test for paired samples, are indicated by asterisks (*). An expression ratio of 1 (no change) is indicated by a red solid reference line, while an expression ratio of 5 (5-fold upregulation) and an expression ratio of 0.2 (5-fold downregulation) are indicated by an orange dashed reference line. Compound #**6** denotes piroxicam, and #**7** denotes meloxicam.

In Caco-2 cells, piroxicam (#**6**), and meloxicam (#**7**), as well as compounds #**1** and #**2**, had no significant impact on *NAG1* at 6 or 24 h, while compound #**3** upregulated the gene expression 9-fold after 6-h treatment without an effect after 24 h. In the case of compounds #**4** and #**5**, the expression of *NAG1* was significantly upregulated, more markedly following the 6-h than 24-h treatment (respectively, 3.4- and 2.3-fold for #**4**, and 4.1- and 1.4-fold for compound #**5**) (Figure 9).

In HCT 116 cells, piroxicam and meloxicam had no significant impact on *NAG1* at 6 or 24 h, while compounds #**1**–**5** upregulated gene expression, respectively, 3.1- and 2.6-fold (#**1**), 2.5- and 5.2-fold (#**2**), 3.1- and 4.7-fold (#**3**), 2.2- and 3.0-fold (#**4**), and 2.1- and 3.6-fold (#**5**) (Figure 9).

**Figure 10:**
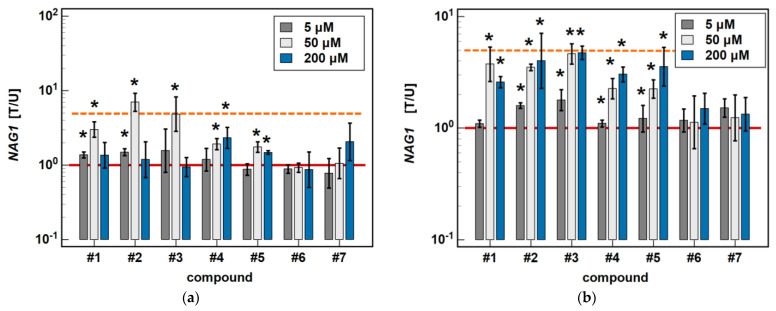
Dose-dependent effect of oxicams on *NAG1* expression in (**a**) Caco-2 cells and (**b**) HCT 116 cells. The bars and whiskers represent the mean ± SD expression ratio of the normalized relative quantities [NRQ] obtained for cells treated with 5, 50, and 200 μM oxicams for 24 h, and the corresponding untreated controls [T/U]. The significant differences between the treated and untreated cells, analyzed using a *t*-test for paired samples, are indicated by asterisks (*). An expression ratio of 1 (no change) is indicated by a red solid reference line, while an expression ratio of 5 (5-fold upregulation) and an expression ratio of 0.2 (5-fold downregulation) are indicated by an orange dashed reference line. Compound #**6** denotes piroxicam, and #**7** denotes meloxicam.

In Caco-2 cells, piroxicam and meloxicam had no significant effect on *NAG1* expression, while compound #**1** upregulated it at 5 and 50 μM (1.4- and 3.0-fold) but not a 200 μM concentration. Likewise, compound #**2** upregulated *NAG1* at 5 and 50 μM (1.5- and 7.0-fold) but not 200 μM, whereas #**3** had a significant stimulatory effect (4-.8-fold) solely at a 50 μM concentration. Compound #**4** upregulated *NAG1* at 50 and 200 μM (1.9- and 2.3-fold, respectively), similarly to #**5** (1.7- and 1.5-fold) (Figure 10).

In HCT 116 cells, piroxicam and meloxicam had no significant effect on *NAG1* expression. Compound #**1** had no effect at 5 μM, but upregulated *NAG1* at 50 and 200 μM concentrations 3.7- and 2.6-fold, respectively. Compound #**2** significantly upregulated the gene expression at 5, 50 and 200 μM, respectively, 1.7-, 3.5- and 5.2-fold. Likewise, compound #**3** upregulated *NAG1* at all of the tested concentrations by 1.8-, 4.6-, and 4–7-fold. Compounds #**4** and #**5** had no effect at 5 μM but significantly and dose-dependently upregulated *NAG1* at 50 and 200 μM 2.3- and 3.0-fold (#**4**), and 2.2- and 3.6-fold (#**5**) (Figure 10).

### 2.5. Effect of Oxicams on NAG1 Protein Expression in Caco-2, HCT 116, and HT-29 Cells

In order to confirm the stimulatory activity of oxicams on NAG1 protein expression and the protein level, Caco-2, HCT 116, and HT-29 were treated with 50 and 200 μM drug concentrations for 48 h, and the cell protein content was analyzed by Western blotting. The HT-29 line was examined additionally, as the Caco-2 cells did not show any reactivity with anti-NAG1 antibodies.

**Figure 11:**
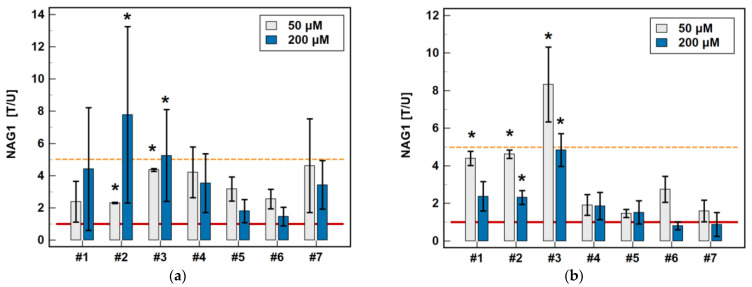
Effect of oxicams on NAG1 protein expression in (**a**) HCT 116 cells and (**b**) HT-29 cells. The bars represent the mean ± SD (whiskers) of the expression ratio between cells treated with 50 or 200 μM oxicams for 48 h, and the corresponding untreated controls [T/U]. The significant differences between the expressions in the treated and untreated cells, analyzed using a *t*-test for one mean, are indicated by asterisks (*). An expression ratio of 1 (no change) is indicated by a red solid reference line, and an expression ratio of 5 (upregulation) is indicated by an orange dashed reference line. Compound #**6** denotes piroxicam, and #**7** denotes meloxicam.

**Figure 12:**
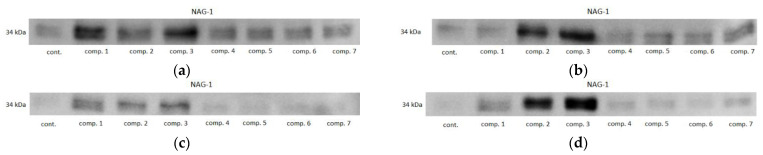
Representative immunoblots of oxicam’s effect on NAG1 protein expression in: (**a**) HCT 116 cells treated with 50 µM oxicams; (**b**) HCT 116 cells treated with 200 µM oxicams; (**c**) HT-29 cells treated with 50 µM oxicams; (**d**) HT-29 cells treated with 200 µM oxicams. Cont., control; comp., compound.

In HCT 116 cells, all of the oxicams at both concentrations upregulated NAG1, but the effect was statistically significant solely for compound #**2**, 2.3-fold at 50 µM and 4.4-fold at 200 µM, and for compound #**3**, 4.4-fold at 50 µM and 7.8-fold at 200 µM. In HT-29 cells, an upregulation was observed for all of the compounds except for 200 µM piroxicam and meloxicam, but statistically significant changes were also noted for compounds #**2** (4.6- and 2.3-fold for 50 and 200 µM, respectively) and #**3** (8.3- and 4.8-fold for 50 and 200 µM, respectively), and for 50 µM of compound #**1** (4.4-fold) (Figure 11). Representative blots are presented in Figure 12.

### 2.6. Effect of Oxicams on the Expression of NFκB-Associated Genes in Caco-2 Cells

In addition, the potential effect of the investigated oxicams on genes encoding proteins associated with NF-κB signaling—namely, *NFKBIA*, *MYD88*, and *RELA*—was examined in Caco-2 cells.

**Figure 13:**
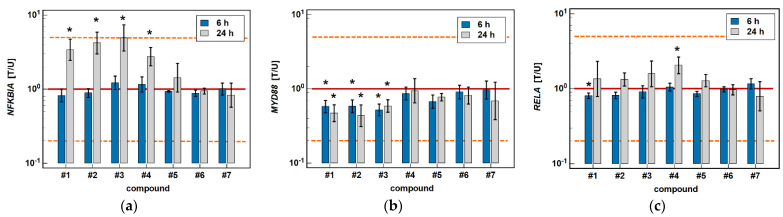
Effect of oxicams on the expression of NFκB-associated genes in Caco-2 cells: (**a**) *NFKB1A*; (**b**) *MYD88*; (**c**) *RELA*. The bars and whiskers represent the mean ± SD expression ratio of the normalized relative quantities [NRQ] obtained for cells treated with 200 μM oxicams for 6 and 24 h, and the corresponding untreated controls [T/U]. The significant differences between the treated and untreated cells, analyzed using a *t*-test for paired samples, are indicated by asterisks (*). An expression ratio of 1 (no change) is indicated by a red solid reference line, while an expression ratio of 5 (5-fold upregulation) and an expression ratio of 0.2 (5-fold downregulation) are indicated by an orange dashed reference line. Compound #**6** denotes piroxicam, and #**7** denotes meloxicam.

The classic oxicams, piroxicam and meloxicam, had no significant effect on any of the investigated NFκB-related genes. In turn, compounds #**1**–**4** significantly upregulated inhibitory *NFKBIA* by 3.4-, 4.2-, 4.9-, and 2.8-fold after 24 h of treatment while having no effect after 6 h. In addition, compounds #**1**–**3** had an inhibitory effect on *MYD88*, both at 6 (1.7-, 1.7-, and 1.9-fold), and 24 h (2.1-, 2.3-, and 1.7-fold). The expression of *RELA* was slightly inhibited by compound #**1** after 6-h treatment (1.2-fold), and was upregulated after 24-h treatment by compound #**4** (2.1-fold) (Figure 13).

### 2.7. Local Expression of MCP and MIP Chemokines in CRC Patients

#### 2.7.1. Pairwise Analysis of MCPs and MIPs in Transformed and Non-transformed Tumor-Adjacent Colorectal Mucosa

The MCP and MIP chemokines displayed different expression patterns, as determined with a reversely-transcribed quantitative polymerase chain reaction (RTqPCR). The expression of *CCL2* and *CCL8* from MCP chemokines and *CCL19* from MIP chemokines was significantly downregulated in tumors compared to patient-matched macroscopically normal tissue by 2.1-fold, 5.1-fold, and 2.2-fold, respectively. The expression of *CCL4* and *CXCL2* from MIP chemokines was significantly, and *CCL3* insignificantly, upregulated in tumors compared to tumor-adjacent non-transformed tissue by 1.4-fold, 2.0-fold, and 1.3-fold, respectively (Table 1).

#### 2.7.2. Non-Paired Analysis of the MCP and MIP Expression in Tumor and Tumor-Adjacent Tissue Compared to Normal Colonic Mucosa from Non-Cancer Patients

In order to establish whether the chemokine expression in tumors is indeed downregulated—or rather, if it is upregulated in tumor-surrounding tissue moreso than in tumors—the chemokine expression in tumors and the adjacent tissue was compared with normal tissue from patients undergoing polypectomy (*n* = 20).

**Figure 14:**
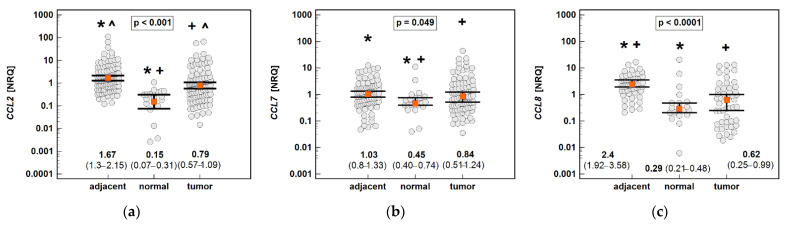
An expression of MCP chemokines in tumor and tumor-adjacent tissue compared to normal mucosa from non-cancer patients: (**a**) *CCL2*; (**b**) *CCL7*; (**c**) *CCL8*. The data are presented as means (*CCL2*) or medians (*CCL7* and *CCL8*) with 95% confidence intervals. The data were analyzed using one-way ANOVA with a Student-Newman-Keuls post hoc test or a Kruskal–Wallis *H* test with a Conover post hoc test, respectively. The significant between-group differences are indicated by the same type of symbol: *, +, or ^. NRQ, normalized relative quantities.

The non-paired analysis confirmed significantly lower *CCL2* and *CCL8* expression in tumors compared to tumor-adjacent tissue. However, compared to the chemokine expression in normal tissue from non-cancer patients, all of the MCPs were significantly upregulated in both tumors and tumor-adjacent tissue. The expression of *CCL2* was upregulated 11.1-fold in tumor-adjacent tissue, and 5.3-fold in tumors compared to normal mucosa, in *CCL7* 2.3- and 1.9-fold, and in *CCL8* 8.3- and 2.1-fold (Figure 14).

**Figure 15:**
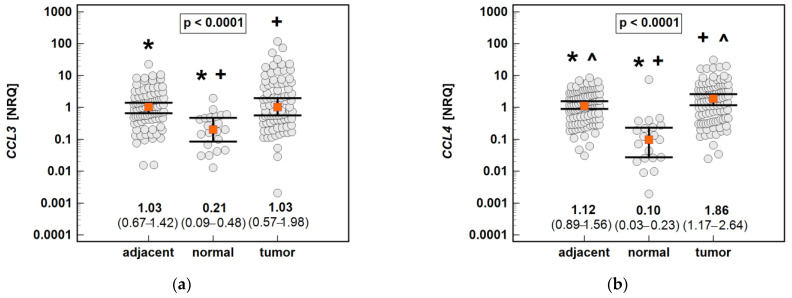

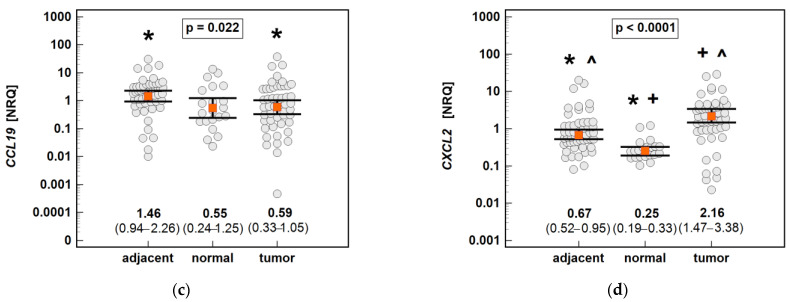
An expression of the MIP chemokines in tumor and tumor-adjacent tissue compared to normal mucosa from non-cancer patients: (**a**) *CCL3*; (**b**) *CCL4*; (**c**) *CCL19;* (**d**) *CXCL2*. The data are presented as means (*CCL19*) or medians (*CCL3*, *CCL4*, and *CXCL2*) with 95% confidence intervals. The data were analyzed using one-way ANOVA with a Student-Newman-Keuls post hoc test or a Kruskal–Wallis *H* test with a Conover post hoc test, respectively. The significant between-group differences are indicated by the same type of symbol: *, +, or ^. NRQ, normalized relative quantities.

The non-paired analysis confirmed significantly higher *CCL19* and significantly lower *CCL4* and *CXCL2* expression in tumor-adjacent tissue than in tumors, and a lack of difference in the case of *CCL3*. Compared to normal tissue from non-cancer patients, the *CCL3*, *CCL4* and *CXCL2* expressions were upregulated in both tumor and tumor-adjacent tissue, while *CCL19* was upregulated solely in tumor-adjacent tissue (Figure 15).

### 2.8. Impact of Cancer Pathology on Chemokine Expression

**Figure 16:**
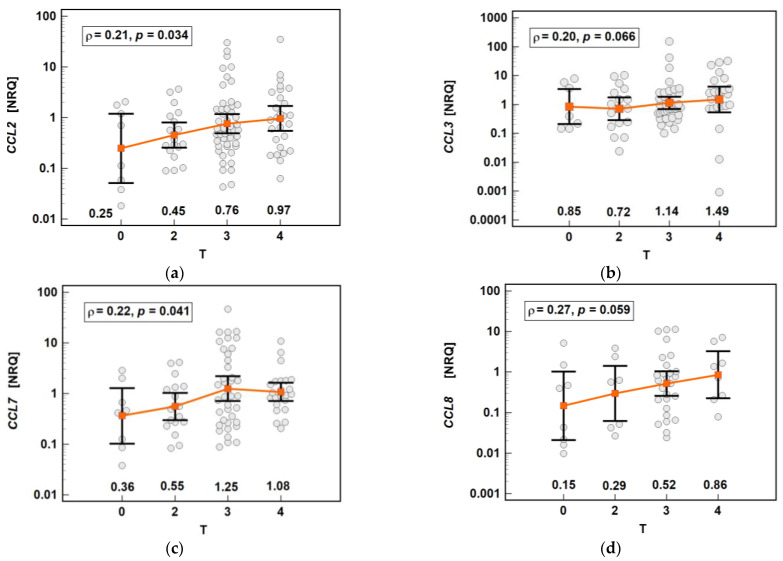
Impact of the depth of tumor invasion on the chemokine expression in tumors: (**a**) *CCL2*; (**b**) *CCL3*; (**c**) *CCL7;* (**d**) *CCL8*. The data are presented as means with 95% confidence intervals, and as Spearman correlation coefficients (*ρ*). NRQ, normalized relative quantities.

The chemokine expression in tumors was dependent on the depth of tumor invasion. The expression of *CCL2* and *CCL7* increased significantly along with the advancing T stage, and that of *CCL3* and *CCL8* increased non-significantly (Figure 16).

**Figure 17:**
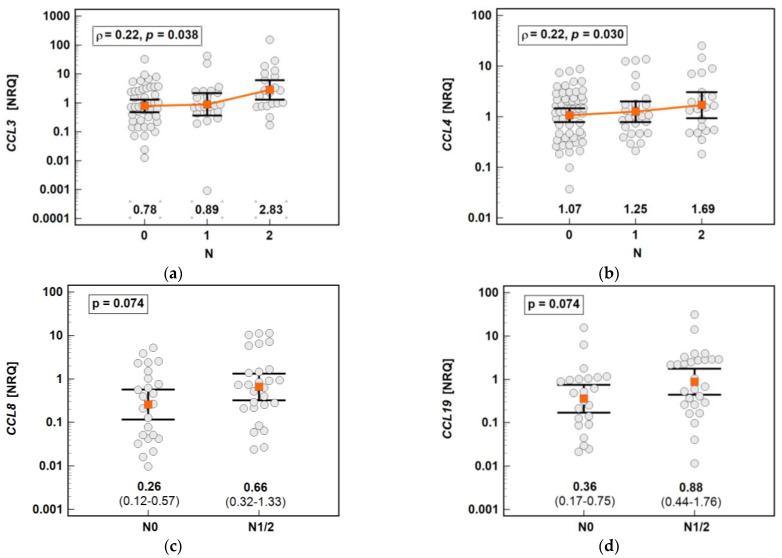
Impact of lymph node metastasis on the chemokine expression in tumors: (**a**) *CCL3*; (**b**) *CCL4*; (**c**) *CCL8;* (**d**) *CCL19*. The data are presented as means with 95% confidence intervals, and as a Spearman correlation coefficient (*ρ*) (*CCL3* and *CCL4*). The data regarding *CCL8* and *CCL19* were analyzed using a *t*-test for independent samples. NRQ, normalized relative quantities.

The chemokine expression in tumors was dependent on lymph node involvement. The expression of *CCL3* and *CCL4* increased along with the advancing N stage (Figure 17). The expression of *CCL8* (2.6-fold) and *CCL19* (2.5-fold) in the tumors of patients with lymph node involvement (N1 or N2) was non-significantly higher than that in the tumors of patients without lymph node metastasis (Figure 16). In the case of *CCL8*, the fold-change in expression (tumor-to-adjacent) in N0 vs. N1/2 patients differed significantly by 3.7-fold (0.1 vs. 0.37, *p* = 0.021).

The local expression of MCPs and MIPs was not dependent on the presence of distant metastases (stage M) or the tumor histopathological grade (G).

The expression of *CCL7* tended to be higher in rectal than colonic tumors by 1.8-fold (*p* = 0.086), without changes in the non-affected mucosa with respect to the tumor location. The *CCL8*, in turn, was significantly (*p* = 0.002) more markedly expressed in non-affected mucosa from right-sided tumors, 2.7-fold compared to rectal tumors and 1.8-fold compared to left-sided tumors.

### 2.9. Interrelationship between the Chemokine Expression in the Bowel and Circulating MCPs and MIPs

In general, the expression of MCP and MIP chemokines was more tightly interrelated in tumor tissue than normal or non-transformed adjacent tissue. The MCPs were more tightly related to each other than to MIPs. The expression of *CCL2* correlated with all of the other chemokines (also in tumor-adjacent tissue), while *CCL19* was the least correlated gene. Except for an inverse relation between *CXCL2* and *CCL19* in non-transformed tumor-adjacent tissue, all of the correlations between the chemokine genes were positive (Table 2).

The local expression of *CCL2*, *CCL3* and *CCL4* was compared to the respective chemokine concentration in the systemic circulation (determined using Luminex xMAP technology). There was a positive association between the *CCL4* expression in tumors and its systemic concentration (*ρ* = 0.35, *p* = 0.022, *n* = 43).

## 3. Discussion

Piroxicam and meloxicam are approved as non-cancer drugs with anti-tumor activity, and are listed in the Repurposing Drugs in Oncology (ReDO) database [31], although the exact mechanisms of their antineoplastic activity are unclear. What seems to be the most important, i.e., the efficacy of piroxicam as a chemopreventive agent, has been demonstrated in animal models [32] as well as in human clinical trials [33]. Herein, we sought to examine its effect on the expression of MCPs and MIPs. Of all of the studied MCP chemokines, the expression of *CCL2* in Caco-2 cells was the highest, while its levels were barely detectable in HCT 116, corroborating the notion of undetectable chemokine expression in another colon adenocarcinoma cell line, LS174T [34]. Unfortunately, the expressions of *CCL7* and *CCL8* were too low for analysis in both cell lines as well. Contrary to novel oxicam analogues, neither piroxicam nor meloxicam had a significant impact on MCP-1/*CCL2* expression or secretion. The *CCL2* in Caco-2 cells displayed an intriguing expression pattern in response to treatment with compounds #**1**–**3**. It was downregulated by 24-h incubation with a 50 μM dose or 6-h incubation with a 200 μM dose, but was substantially upregulated by 24-h incubation with a 200 μM dose. Such a biphasic response may imply the involvement of two different regulatory mechanisms: one operating at a low dose or shorter exposure, and the other at a high dose and longer exposure. Importantly, the observation was confirmed at the protein level and mimicked by *CXCL2*, an MIP chemokine, which was also measurable in HCT 116. Like piroxicam and meloxicam, compounds #**4** and #**5** had no significant impact on MCP-1/*CCL2* expression and secretion, except the inhibitory effect of compound #**5** on gene expression in Caco-2.

Structurally, studied novel analogues are distinguished from classic oxicams by two substitutions in the thiazine ring of the oxicam scaffold: arylpiperazine pharmacophore at position 2 replacing the methyl substituent at position 2, and a benzoyl moiety replacing 2-peridocarbamoyl at position 3. Arylpiperazine has high electron-withdrawing properties, which translate into enhanced anti-inflammatory properties [35]. While an upgraded anti-inflammatory activity may explain the inhibitory effect of oxicam analogues on MIP-1α/*CCL3* and MIP-1β/*CCL4* observed for all of the novel compounds, it does not clarify the selective effect of #**1** to **3** on MCP-1/*CCL2*. Compounds #**1** to **3** differ from non-effective compounds #**4** and **5** with a linker between the thiazine and arylpiperazine rings. Our results imply that a three-carbon propylene linker is crucial for the modulation of MCP-1/*CCL2* expression and secretion, while the two-carbon oxyethylene linker present in compounds #**4** and **5**, shown to enhance analgesia [36], is ineffective. Compounds #**2** and #**3** were more effective than compound #**1**, indicating the significance of electron-withdrawing fluoro-substituents at the arylpiperazine pharmacophore (compounds #**2** and #**3**), and the benzoyl moiety (compound #**3**), which are absent in compound #**1**. Regarding the upregulation of MCP-1/*CCL2* in response to a high oxicam dose and longer exposure, the expression of MCP-1 has been shown to be upregulated several-fold in ovarian cancer by chemotherapy, and the effect has been reproducible at the mRNA and protein levels, both in vitro and in vivo [34]. Likewise, radiation has been demonstrated to induce MCP-1 secretion from breast cancer cells [37]. Interestingly, the MCP-1 response to chemotherapy could be mimicked by the inhibition of the JAK/STAT signaling pathway, implying a suppressive role of this pathway towards MCP-1 [34]. Therefore, the overexpression of JAK/STAT in colorectal tumors, reported elsewhere and positively associated with cancer advancement and poor prognosis [38], may explain the lower *CCL2* expression in tumors compared to tumor-adjacent tissue observed in the present study. In turn, the inhibitory effect of NSAIDs on JAK/STAT signaling, potentially alleviating their suppressive activity towards MCP-1, might explain the upregulating effect of compounds #**1** to **3** on MCP-1/*CCL2*. It is also possible that MCP-1/*CCL2* overexpression is a part of the stress response, especially given that these particular oxicam analogues are effective inducers of apoptosis and cell cycle arrest (unpublished results). They have also induced such a biphasic response in colonic cell lines regarding arginase-2 expression from the L-arginine/nitric oxide pathway [25].

The expression and secretion of MIP-1α/*CCL3* and MIP-1β/*CCL4* were only non-significantly decreased by the classic oxicams, with the exception of a 9.9-fold downregulation of *CCL4* in Caco-2 cells and a weak downregulation of *CCL3* in HCT 116 by meloxicam. In turn, MIP-1α/*CCL3* and MIP-1β/*CCL4* were consistently downregulated by novel oxicam analogues. Compounds #**1** to **5** inhibited the MIP-1α/*CCL3* and MIP-1β/*CCL4* expression in both Caco-2 and HCT 116 cells, and secretion in Caco-2. Intriguingly, compound #**3** suppressed *CCL3* expression dose- and time-dependently solely in HCT 116, but consistently had no effect in Caco-2 cells, either on chemokine expression or secretion. It has also failed to reduce the *CCL4* expression in Caco-2, which, owing to too-low gene expression, could not be confronted with HCT 116. However, the lack of effect at the transcript level did not translate to the protein level, as compound #**3** reduced MIP1β/*CCL4* secretion by 30%.

The inhibitory effect on MIP-1α/*CCL3* and MIP-1β/*CCL4* was the strongest and the most consistent in the case of compound #**2**. This may imply the superiority of three-carbon propylene linker between thiazine and arylpiperazine nitrogen atoms over the oxoethylene linker present in compounds #**4** and #**5**. It may also indicate that one electron-withdrawing substituent is more effective than none (compound #**1**) or two such substituents (compound #**3**). The impact of the studied oxicams on *CXCL2* expression resembled the effect observed for *CCL2*. The gene expression in Caco-2 was downregulated during a 6-h incubation with a 200-μM dose of compounds #**1** to **3** and a longer exposure to 50 μM compound #**2**, but was upregulated by a 200-μM dose of #**2** and #**3** upon 24-h exposure. In HCT 116, compounds #**1** to **3** had a positive time- and dose-dependent effect on *CXCL2* expression without an inhibitory impact at lower doses or upon shorter exposure. The exception was a slight inhibition by the 5 μM compound #**1**. As MIP-2/*CXCL2* was not included in the cytokine panel, the effect could not be confirmed on the protein level. In addition, compound #**5** inhibited *CXCL2* expression at 50 and 200 μM. The literature data on MIP-2/*CXCL2*, also known as GRO-2 (or β), are scanty and confusing concerning its cancer-related up- or downregulation [39,40] or activity [41]. Nonetheless, the cumulative evidence ascribes MIP-2 a pro-tumorigenic role as a proangiogenic factor and a potent chemoattractant for neutrophils and MDSCs, involved in cancer-related immunosuppression. Moreover, only recently, soluble MIP-2 has been proposed as a marker of the treatment response to immune checkpoint inhibitor therapy against programmed death (PD)-1 in advanced or recurrent non-small-cell lung cancer [42]. Although an early study [43] found the *CXCL2* expression in normal and neoplastic colonic tissue to be only minimal, subsequent analyses of McLean et al. [44] and Doll et al. [45] demonstrated significant chemokine upregulation in colonic adenocarcinomas.

In addition to being COX inhibitors, NSAIDs, including oxicams, have been shown to operate via mechanisms independent of the COX/prostaglandin E pathway. Among others, they have been shown to induce the expression of the NSAID-activated gene (NAG1), which is also known as a growth differentiation factor (GDF)-15 or macrophage inhibitory cytokine (MIC)-1 [46]. NAG1/GDF-15/MIC-1 belongs to a TGF-β super-family, and controls the expression of several inflammatory mediators, including MCP chemokines [47]. Its activation has been linked with a reduced propensity to develop intestinal polyps and tumors in mice (reviewed in [48]), which, combined with its expression being regulated by NSAIDs, evoke an interest in NAG1/GDF-15/MIC-1 as a chemopreventive agent. Therefore, we examined the oxicam effect on NAG1 and demonstrated that novel oxicams, but not classic drugs, stimulate its expression. The induction of *NAG1* was observed in both COX-expressing (Caco-2), and non-expressing (HCT 116) cell lines, although the effect seemed to be more marked in HCT 116 cells. The greater responsiveness of HCT 116 observed in the present study is consistent with the inverse relationship between NAG1 and COX expression reported elsewhere [49]. All of the studied compounds, to a varying degree, upregulated *NAG1*, although compounds #**2** and #**3** seemed to be slightly more effective, indicating the significance of both the propylene linker between thiazine and arylpiperazine nitrogen atoms and fluoride substituents at arylpiperazine and benzoyl moieties. Compounds #**2** and #**3** were also the ones to significantly upregulate the NAG1 protein in HCT 116 and HT-29 cells. The ability of novel oxicam analogues to act by inducing NAG1 expression is of particular clinical relevance in regard to IBD patients, both in terms of chemoprevention and mucosal healing. Recently, a muco-protective role in the gastrointestinal tract was attributed to NAG1/GDF-15/MIC-1, as it has been shown to promote early epithelial restitution following intestinal injury [50]. As such, the upregulation of NAG1 expression observed in this study might be one of the molecular mechanisms behind the declared reduced gastrointestinal toxicity of novel oxicam analogues [24].

As Caco-2 cells displayed no NAG1 immunoreactivity, we examined oxicam’s effect on their expression of *NFKBIA*, *MYD88* and *RELA*, i.e., genes associated with NF-κB signaling, known to control MCP and MIP expression as well as being regulated by NSAIDs. The analogues characterized by the presence of the three-carbon propylene linker (compounds #**1**–**3**), but not classic oxicams, substantially upregulated the NF-κB inhibitor (*NFKBIA*) while downregulating an adaptor protein (*MYD88*). The effect was strengthened by the presence of fluoro-substituents at the arylpiperazine and benzoyl moieties.

Leukocyte-attracting chemokines are key mediators of inflammation, displaying a number of pro-neoplastic activities [2,3,4,5]. However, there is a paucity of clinical reports regarding MCP and MIP chemokines, especially those conducted on representative populations/sample sets, although data derived from in vitro and animal studies are rapidly accumulating. In this study, we showed that none of the evaluated MCP chemokines were upregulated in tumors compared to non-transformed tumor-adjacent tissue. Similar results, obtained in an RNA-Seq analysis of five patient-matched samples of normal mucosa, adenoma, and carcinoma have recently been published [51]. In fact, the expression of *CCL2* and *CCL8* was downregulated in the present study two- and five-fold, respectively. Although surprising at first, it transpired not to be synonymous with chemokine downregulation in cancer. As revealed by comparison with colonic mucosa from non-cancer individuals, both transformed and non-transformed tissue from CRC patients had upregulated *CCL2* (5.3- and 11.1-fold, respectively), *CCL7* (1.9- and 2.3-fold), and *CCL8* (2.1- and 8.3-fold), although non-significantly in the case of tumor *CCL8*. Moreover, the expression of all of the MCPs increased along with the advancing T stage and a fold-change in *CCL8* expression (tumor-to-adjacent) was higher by 3.7-fold in patients with lymph node involvement. Corroborating our observation of *CCL2*′s association with tumor progression, the limited reports on MCPs in CRC have indicated, albeit not unanimously [52], an increase in *CCL2* expression along with an advancing clinical stage, which has been accompanied by the accumulation of TAMs [53,54]. The particularly close association of MCP with metastatic CRC [55,56] has been indicated in functional studies, in which tumor-derived MCP-1 enabled efficient cancer cell extravasation [55] and mediated the stimulatory effect of alcohol on invasion and metastasis [57].

Non-transformed tumor-surrounding tissue is not passive, and has repeatedly been shown to have an altered molecular landscape [25,58,59,60] despite the lack of morphological and histological changes. Pre-existing cancer-initiating changes in apparently normal mucosa are believed to contribute to the failure of surgical treatment and local tumor recurrence, as well as to the occurrence of synchronous tumors [61]. The upregulation in tumors and in the adjacent tissue may serve different purposes, as was suggested for IL32, the overexpression of which in gastrointestinal tumors reflected proliferative and metastatic potential, and antiapoptotic and immunosuppressive capacity in non-transformed mucosa [58]. Like IL32, the expression of *CCL8* was upregulated in the non-transformed tissue of right-sided cancers, which are known to be more aggressive. This may imply the contribution of *CCL8* to the molecular mechanism behind the higher recurrence rates of right- compared to left-sided cancers [62]. Moreover, the existence of the *CCL8* gradient, that is, an increasing concentration from neoplastic epithelium via stroma to the periphery, has been reported and found to be a driving force of breast cancer metastasis [63]. The significance of MCP-1 expression in non-transformed mucosa has been demonstrated in an animal model of colon carcinogenesis employing MCP-1 KO mice, in which gene deletion downregulated the expression of proinflammatory cytokines and upregulated the suppressor of cytokine signaling (SOCS)-1 in both tumors and the adjacent tissue [64].

The role of MCP-3 in cancer seems to be ambiguous. On one hand, transfection with *CCL7* has been shown to retard or completely inhibit tumor growth and prevent formation of metastasis, on the other–MCP-3 facilitates the formation of an environment favoring carcinoma progression and promotes phenotypic transformation in monocytes (reviewed in [65]). Clinically, the association of *CCL7*/MCP-3 with CRC development has been demonstrated by an over 9-fold up-regulation of its expression in liver-metastases compared to patient-matched primary tumors [66].

Unlike MCPs, the MIP chemokines were upregulated in tumors compared to non-transformed adjacent tissue, although *CCL3* overexpression was marginal and non-significant. They were also overexpressed in both tumors (*CCL3* by 4.9-fold, *CCL4* by 18.6-fold, and *CXCL2* by 8.6-fold), and adjacent tissue (*CCL3* by 4.9-fold, *CCL4* by 11.2-fold, and *CXCL2* by 2.7-fold) compared to normal mucosa. The exception was *CCL19*, expressed at a similar level in tumor and normal tissue, and significantly downregulated in tumors compared to normal mucosa. The overexpression of MIP was associated with lymph node metastasis. Unlike with other chemokines, there seems to be a consensus on the anti-tumor role played by MIP-3β/*CCL19,* at least in CRC. Its downregulation observed in this study corroborates the findings of others regarding chemokine transcripts [67] as well as protein [67,68]. The *CCL19* gene has also been listed among 10 hub genes identified by integrated bioinformatics analysis as having high diagnostic potential for the detection of CRC, in which it was consistently down-regulated [69]. Functional studies have shown the anti-tumor activity of MIP-3β, resulting from its ability to increase the number of anti-tumor immune cells [70], to suppress angiogenesis [68], and to increase the expression and secretion of IFN-γ and IL-12 [71].

The MIP-1α/*CCL3* and MIP-1β/*CCL4* chemokines may display either anti-tumor or tumor-promoting activity, and the available data seem to support both roles in equal measures. An upregulation of MIP-1α and MIP-1β proteins in colorectal tumors [72] and chemokine elevation in the circulation was associated with disease advancement [73] and a worse prognosis [74]. The *CCL4* expression is higher in patient-matched liver metastases than primary tumors [56]. Functional studies have attributed MIP-1α an angiogenesis-promoting activity [75], thus corroborating the association of *CCL3* with lymph node involvement demonstrated in the present study. MIP-1α/*CCL3* exacerbates immune-mediated colitis [76], and gene deletion reduces colonic tumorigenesis [77]. Nonetheless, there seems to be equally strong evidence of the anti-tumor activity of MIP-1α and β in CRC. In a large prospective study, a low MIP-1α concentration has been associated with an increased risk for CRC [65]; in another one, increased serum concentrations of MIP-1β were found to predict improved disease-free survival [78]. A comparative analysis of the gene expression patterns between CRC microsatellite-instable (MSI) and microsatellite-stable tumors indicated that *CCL3* and/or *CCL4* were more strongly expressed in MSI. Indirectly, this links both genes with a favorable prognosis, as MIS in CRC is associated with prolonged survival and lower recurrence rates [79]. Functional studies support the anti-tumor activity of MIP-1α and β in CRC as well. MIP-1α/*CCL3* has inhibited the progression of established tumors by the preferential recruitment of anti-tumor immune cells and the production of anti-tumor chemokines [80]. Correspondingly, *CCL4* gene transfer has inhibited tumor growth and prolonged the survival of tumor-bearing mice [81].

## 4. Materials and Methods

### 4.1. Patients

#### 4.1.1. Study Population

Enrolled patients with histologically confirmed adenocarcinomas of the colon or rectum were admitted to the Department of Surgical Oncology, Regional Hospital in Wroclaw (2013–2015) or the First Department and Clinic of General, Gastroenterological and Endocrinological Surgery of Wroclaw Medical University (2012–2013) for curative tumor resection. The patients were subjected to the routine preoperative workup, which included colonoscopy, abdominal and pelvic CT, and a pelvic MRI for rectal cancer. The exclusion criteria included: age < 18 years, ASA physical status classification system >3, emergency surgery, locally advanced cancers which were not amenable to curative resection or gross metastatic disease, tumors requiring *en bloc* multi-visceral resection, the presence of other synchronous cancers, severe cardiovascular or respiratory disease, severe mental disorders, and immunological diseases requiring the systemic administration of corticosteroids. The resected tumors were staged using the Union for International Cancer Control (UICC) tumor-node-metastasis (TNM) system. The patients’ characteristics are summarized in Table 3. As the analyzes were conducted on biobanked material, the cDNA template was not always available for all of the quantified genes. Therefore, the patients’ characteristics are presented separately for the *CCL2*/*CCL4*, *CCL3*/*CCL7* and *CCL8*/*CCL19*/*CXCL2* cohorts.

#### 4.1.2. Biological Material

The tissue samples from the cancer patients were obtained intraoperatively, washed in PBS, and preserved in RNAlater solution (Ambion Inc., Austin, TX, USA). Normal large bowel samples were obtained from patients undergoing polypectomy and preserved in RNAlater solution as well. Only samples from patients whose polyps were subsequently verified through histopathological examination as hyperplastic polyps or tubular adenomas, thus having the lowest potential for malignancy, were used in the current study as a reference (*n* = 20). All of the tissue samples were kept frozen at −80 °C until the RNA isolation.

### 4.2. In Vitro Studies

#### 4.2.1. Oxicams

Piroxicam (compound #**6**), and meloxicam (compound #**7**) were obtained from commercial sources: Sigma-Aldrich (St. Luis, MO, USA) and Alfa Aesar (Thermo Fisher Scientific, Waltham, MA, USA), respectively, and served as reference standards. The novel oxicam analogues, denoted as compounds #**1**–**5**, were synthesized as previously described [82,83,84]. In short, saccharin (1,1-dioxo-1,2-benzothiazol-3-one), as a starting material, was condensed in dimethylformamide (DMF), and in the presence of triethylamine (TEA) with one of the following: 2-bromoacetophenone (in the case of compound #**1**), 2-bromo-4′-fluoroacetophenone (in the case of compounds #**2**, #**3** and #**4**), or 2-bromo-4′-chloroacetophenone (in the case of compound #**5**). The resulting condensation products were subsequently rearranged to the corresponding 1,2-benzothiazine ring in a Gabriel–Colman rearrangement. The final compounds were prepared by the alkylation of the corresponding 1,2-benzothiazine with 1-(3-chloropropyl)-4-phenylpiperazine (in the case of compounds #**1** and #**2**) or 1-(3-chloropropyl)-4-(2-fluorophenyl)piperazine (in the case of compound #**3**), or with 1-(2-chloroacetyl)-4-(2-fluorophenyl)piperazine (in the case of compounds #**4** and #**5**). The resulting products were separated and purified by crystallization from ethanol. The compounds’ structures, presented in Table 4, were confirmed by elemental and spectral analyses (MS, FTIR, 1H NMR, 13C NMR).

#### 4.2.2. Cell Cultures

Certified human colon cancer adherent epithelial cell lines—Caco-2 (ATCC^®^ HTB-37™), HCT 116 (ATCC^®^ CCL-247™), and HT-29 (ATCC^®^ HTB-38™)—purchased from the American Type Culture Collection (ATCC; Manassas, VA, USA) were cultured in Dulbecco’s Modified Eagle Medium (DMEM; without phenol red, 25 mM glucose; Gibco, Thermo Fisher Scientific) supplemented with 10% heat-inactivated fetal bovine serum (FBS; Gibco), and 1% stabilized antibiotic antimycotic solution containing 10,000 units of penicillin/mL, 10 mg/mL of streptomycin, and 25 µg/mL of amphotericin B (Sigma Aldrich). The culture medium was renewed twice per week. The cells were cultured in T-75 cell culture flasks (Eppendorf AG, Hamburg, Germany) at 37 °C in humidified air containing 5% CO_2_, in a CELCULTURE^®^ CCL-170B-8 incubator (Esco Micro Pte Ltd., Singapore). The cells were passaged after reaching approximately 80% confluency, preceded by harvesting with TrypLE™ Express (Gibco, Thermo Fisher Scientific), staining with 0.4% trypan blue solution, and counting with the Countess™ Automated Cell Counter (Invitrogen, Thermo Fisher Scientific, CA, USA).

For the transcriptomic analysis, the chemokine profiling (Caco-2 and HCT 116) and the protein analysis (HT-29, HCT 116 and Caco-2) cells were seeded at 2 × 10^5^ cells per well on 6-well plates (Eppendorf AG), and cultured for 24 h at 37 °C, 5% CO_2_, followed by 6- and 24-h (transcriptomic analysis and chemokine profiling) or 48-h (protein analysis) treatment with the tested compounds, i.e., meloxicam, piroxicam, or novel analogues of oxicams: compounds #**1**–**5** dissolved in dimethyl sulfoxide (up-to 0.5%; DMSO) at 5, 50 and 200 µM for transcriptomic analysis and chemokine profiling, or 50 and 200 µM for the protein analysis. Cells cultured with DMEM 10% FBS were used as the controls. Due to its lower solubility, compound #**1** at 200 µM was dissolved in 1% DMSO. Cells treated with 1% DMSO were used as a respective control. After the treatment, post-culture media were collected for the chemokine profiling. For the transcriptomic analysis, preceded by RNA isolation, the cells were lyzed with TRIzol™ Reagent (Thermo Fisher Scientific). For the protein analysis, the post-culture media were discarded, the cells were rinsed twice with phosphate-buffered saline (PBS), and were mechanically detached after the addition of a cocktail of protease inhibitors (Complete Tablets EDTA-free; Roche Diagnostics, Mannheim, Germany) in PBS. The collected post-culture media and cell lysates and suspensions were kept frozen at −80 °C for further analysis.

The oxicam dose and the length of treatment were chosen based on literature data and the previously published results of a sulforhodamine B (SRB) assay [25].

### 4.3. Analytical Methods

#### 4.3.1. Transcriptomic Analysis

Paired samples of affected and non-affected colorectal mucosa (up-to 40 mg), harvested postoperatively from CRC patients, were added into a lysis buffer (Invitrogen) supplemented with 2-mercaptoethanol (100:1) (Sigma-Aldrich), and were homogenized in a Fastprep 24 Homogenizer (MP Biomedical, OH, USA) using dedicated ceramic spheres.

A phenol-chloroform extraction was applied to isolate RNA from the clinical samples and cell culture lysates. The isolated RNA was subsequently purified using a PureLink™ RNA Mini Kit (Invitrogen). Potential contamination with genomic DNA was avoided owing to the on-column treatment with DNase (PureLink™ DNase Set, Invitrogen). The purified RNA was quantified using a NanoDrop 2000 (Thermo-Fisher Scientific) with the concomitant evaluation of RNA purity (absorbance ratios: 260/280 nm and 260/230 nm). The RNA integrity, in turn, was assessed with LabChip microfluidic technology on the Experion platform, using dedicated Experion RNA StdSens analysis kits (BioRad, Herkules, CA, USA).

Aliquots of 500 ng or 1000 ng RNA from clinical samples or cell cultures, respectively, were reversely transcribed following the manufacturer’s instructions with an iScript™ cDNA Synthesis Kit (BioRad).

Polymerase chain reactions were conducted on CFX96 Real-Time PCR thermocycler (BioRad) using SsoFast EvaGreen^®^ Supermix (BioRad) and the following cycling conditions: 30 s activation at 95 °C, 5 s denaturation at 95 °C, annealing/extension for 5 s at 61 °C, 40 cycles, followed by a melting step (60–95 °C; fluorescent reading every 0.5 °C) to assure product specificity. The reaction mixture included 2 µL diluted (1:5) cDNA, 1 µL each of 10 nM forward and reverse target-specific primers (Genomed, Warsaw, Poland), 10 µL 2 × SsoFast EvaGreen^®^ Supermix (BioRad), and water up to 20 µL. The primers’ sequences are presented in Table 5.

The technical replicates were averaged. The geometric mean of all of the Cq values in a given sample set was calculated. Individual sample Cq values were subtracted from this mean, yielding ΔCq, and subsequently linearized by a 2^ΔCq^ conversion and normalized to the geometric mean of *PPIA* and *RPLP0* (in the case of clinical samples), or to *GAPDH* (in the case of the cell culture experiments). The resulting values are referred to as a normalized relative quantity (NRQ) [85] and subjected to statistical analysis. The reference gene selection was based on our previous research indicating that *PPIA* and *RPLP0* are the most suitable pair of genes for studies on bowel tissues from CRC patients [86], while *GAPDH* is stably expressed in Caco-2 and colonic adenocarcinoma cell lines under stress-inducing conditions [87].

#### 4.3.2. Protein Analysis

The cell suspensions were sonicated on ice in two 30 s cycles with 40% amplitude using the ultrasonic processor UP 200 (Hielscher Ultrasound Technology, Teltow, Germany). Cell debris was removed by centrifugation (12,500× *g*, 10 min at 4 °C). The total protein concentration was measured colorimetrically at λ = 562 in the supernatants using the bicinchoninic acid assay (Thermo Fisher Scientific, Waltham, MA, USA) with an Infinite^®^ M200 plate spectrophotometer (Tecan Group Ltd., Männedorf, Switzerland). Protein samples (10 µg) were diluted 1:1 with 2 × Laemmli sample buffer (Bio-Rad, Hercules, CA, USA) containing 5% 2-mercaptoethanol, denatured (5 min at 95 °C), and resolved by SDS-PAGE electrophoresis. Gels (0.75 mm, 4% stacking gel and 10% resolving gel) were run using Mini-Protean Tetra Cell (Bio-Rad) at a constant voltage (150 V). Subsequently, the separated proteins were transferred (5 min at the constant voltage of 25 V) with the Trans-Blot Turbo transfer system (Bio-Rad) to the Immobilon P membrane (Merck-Millipore, Burlington, MA, USA). A dedicated transfer buffer (25 mM Tris, 190 mM glycine, 20% methanol) was used. After the transfer, the membranes were stained with Pierce™ Reversible Protein Stain Kit for PVDF Membranes (Thermo Fisher Scientific) for the total protein according to the manufacturer’s instructions. Thereafter, the membranes were blocked with 1% casein blocking buffer (Sigma-Aldrich, Saint Louis, MO, USA) at room temperature for 1 h. After rinsing with PBS-T buffer consisting of PBS (VWR International, Radmor, PA, USA) and 0.05% Tween-20 (Thermo Fisher Scientific, Waltham, MA, USA), the membranes were incubated (overnight, at 4 °C) with specific primary antibodies against NAG-1 (sc-3771961; Santa Cruz Biotechnology, Dallas, TX, USA) diluted 1:200 in PBS-T buffer pH 7.3. The unbound antibodies were removed by washing with PBS-T (3 times, 5 min). Subsequently, the membranes were incubated with HRP-conjugated goat anti-mouse secondary antibodies (Dako A/S, Glostrup, Denmark, cat. no. P044701-2) at a dilution of 1:20,000. The NAG-1 protein bands were visualized using an Immobilon ECL Ultra Western HRP Substrate (Merck-Millipore, Burlington, MA, USA) and ChemiDoc MP Imaging System (Bio-Rad). The obtained Western blot membrane images were analyzed with Image Lab software version 6.0.1 build 34 (Bio-Rad). The intensity of the chemiluminescence signal was normalized to the total protein signal intensity recorded for individual lanes.

#### 4.3.3. Chemokine Profiling

For the purpose of the correlation analysis, the previously published [88,89] data on circulating chemokines were obtained from the patients’ database. In the original research, concentrations of MCP-1, MIP-1α and MIP-1β were measured in sera in duplicates/triplicates using a flow cytometry-based method with a BioPlex 200 platform (Bio-Rad) and Bio-Plex Pro™ Human Cytokine, Chemokine, and Growth Factor Magnetic Bead–Based Assays, as instructed by the manufacturer.

For the purpose of this study, the concentrations of MCP-1, MCP-3, MIP-1α, and MIP-1β were measured in cell culture supernatants using the same methodology and a custom-made cytokine panel ProcartaPlex Immunoassay from Invitrogen (Thermo Fisher Scientific).

Standard curves were prepared using 5-PL logistic regression, and the collected data were analyzed using BioPlex Manager 6.0 software.

### 4.4. Statistical Analysis

The statistical analysis was conducted with MedCalc^®^ Statistical Software version 20.008 (MedCalc Software Ltd., Ostend, Belgium). The Kolmogorov–Smirnov and Levene tests were applied to test the data distribution and homogeneity of variances, respectively. The RTqPCR data were analyzed as logarithms. Matched tissue samples and treated–untreated cells were analyzed using a *t*-test for paired samples. Multigroup comparisons were conducted with one-way ANOVA followed by a Student–Newman–Keuls post hoc test for normally distributed data with homogeneous variances, or with a Kruskal–Wallis *H* test followed by a Conover post hoc test for non-normally distributed data or/and with non-homogeneous variances. Two-group comparisons of unpaired samples were conducted using a *t*-test for independent observations. The correlation analysis was conducted using a Spearman rank correlation yielding correlation coefficient rho (*ρ*) or a Pearson correlation yielding correlation coefficient *r*. The significance of altered chemokine secretion compared to the control cells was tested using a *t*-test for one mean. A probability (*p*) equal to or below 0.05 (two-sided) was considered to be indicative of the statistical significance of observed differences or trends.

## 5. Conclusions

Newly synthesized analogues of the oxicam class of NSAIDs labeled with reduced gastrotoxicity, differing from piroxicam and meloxicam with an arylpiperazine moiety at the thiazine ring, are able to downregulate MCP and MIP chemokines to varying degrees, while the classic oxicams had a mostly negligible or non-significant effect on their expression and secretion. The presence of a propylene linker between thiazine and piperazine nitrogen atoms and one fluorine substituent at arylpiperazine pharmacophore characterizes their most effective and consistent inhibitor. Propylene linker-possessing analogues elicit a biphasic response in terms of *CCL2* and *CXCL2* expression, implying that these analogues might operate by two different mechanisms: an anti-inflammatory mechanism at lower doses and shorter exposures, and evoking a stress response at higher concentrations and longer exposures.

The NSAID-activated gene (NAG1), and *NFKBIA* and *MYD88* from the NFkB signaling pathway are molecular targets of the investigated oxicams located upstream of chemokines. As is consistent with findings regarding chemokines, the analogs with a three-carbon propylene linker are more effective in the upregulation of *NFKBIA* and the downregulation of *MYD88* than oxicams with a two-carbon oxyethylene linker.

Colorectal cancer is associated with aberrantly expressed MCP and MIP chemokines. They are all, with the exception of *CCL19*, upregulated in both transformed and non-transformed mucosa from cancer patients compared to normal tissue from non-cancer patients. Interestingly, while the upregulation in tumors is markedly higher for *CCL4* and *CXCL2*, the *CCL2*, *CCL8* and *CCL19* transcripts are relatively downregulated in tumors compared to tumor-adjacent tissue. High chemokine expression in morphologically and histologically normal mucosa is clinically relevant, as it may predispose to neoplastic transformation and contribute to local cancer recurrence following surgery, or may drive an occurrence of synchronous tumors.

## Figures and Tables

**Table 1:** Pairwise analysis of the expression of MCP and MIP chemokines in the colorectal mucosa from CRC patients.

Chemokines	Gene	*N*	Adjacent [NRQ]	Tumor [NRQ]	Fold Change in Tumors	*p* Value
MCP	*CCL2*	101	1.44 (1.11–1.87)	0.68 (0.50–0.91)	↓ 2.1 (1.5–3.1)	0.0001
	*CCL7*	86	0.90 (0.70–1.17)	0.91 (0.66–1.25)	1 (0.7–1.4)	0.963
	*CCL8*	51	2.13 (1.64–2.75)	0.42 (0.25–0.71)	↓ 5.1 (2.8–9.0)	<0.0001
MIP	*CCL3*	86	0.82 (0.61–1.10)	1.09 (0.74–1.60)	↑ 1.3 (0.9–2.0)	0.171
	*CCL4*	101	0.87 (0.71–1.07)	1.23 (0.97–1.55)	↑ 1.4 (1.1–2.0)	0.022
	*CCL19*	51	1.29 (0.85–1.94)	0.58 (0.35–0.96)	↓ 2.2 (1.3–3.8)	0.005
	*CXCL2*	51	0.75 (0.54–1.05)	1.50 (0.95–2.36)	↑ 2.0 (1.3–3.10)	0.003

The data are presented as geometric means with a 95% confidence interval, and were analyzed with a *t*-test for paired samples. The fold-change in tumor tissue compared to the adjacent non-transformed tissue is marked as decreased (↓) or increased (↑). *N*, number of paired observations available for analysis; NRQ, normalized relative quantity.

**Table 2:** Interrelationships between the expression of MCPs and MIPs.

Gene		*CCL3*	*CCL4*	*CCL7*	*CCL8*	*CCL19*	*CXCL2*
*CCL2*	N	ns	0.78 ^1^	ns	0.60 ^3^	ns	ns
	A	0.30 ^3^	0.54 ^1^	0.36 ^2^	0.35 ^3^	ns	0.55 ^1^
	T	0.44 ^1^	0.57 ^1^	0.76 ^1^	0.89 ^1^	0.46 ^2^	0.36 ^3^
*CCL3*	N		ns	ns	ns	ns	ns
	A		0.44 ^1^	ns	ns	ns	0.47 ^2^
	T		0.56 ^1^	0.37 ^2^	0.69 ^1^	0.33 ^4^	0.53 ^1^
*CCL4*	N			0.58 ^3^	0.66 ^3^	ns	ns
	A			0.25 ^4^	ns	ns	0.36 ^3^
	T			0.53 ^1^	0.49 ^2^	0.45 ^2^	ns
*CCL7*	N				0.57 ^3^	ns	ns
	A				0.27 ^4^	ns	0.27 ^4^
	T				0.83 ^1^	0.29 ^4^	0.39 ^3^
*CCL8*	N					ns	ns
	A					ns	ns
	T					0.40 ^3^	0.40 ^3^
*CCL19*	N						ns
	A						−0.40 ^3^
	T						ns

The data are presented as Pearson correlation coefficients (*r*). ^1^ *p* ≤ 0.0001; ^2^ *p* ≤ 0.001; ^3^ *p* ≤ 0.01; ^4^ *p* ≤ 0.05. ns, non-significant; N, normal colonic mucosa from non-cancer patients; A, tumor-adjacent tissue; T, tumor.

**Table 3:** Characteristics of the patients with colorectal adenocarcinomas.

Parameter	Characteristics		
	*CCL2/CCL4*	*CCL3/CCL7*	*CCL8/CCL19/CXCL2*
Number of patients, *n*	101	86	51
Sex distribution [F/M], *n*	37/64	31/55	21/30
Age [years], mean (95% *CI*)	67.4 (65.3–69.5)	67.5 (65.3–69.7)	67.5 (64.5–70.5)
Cancer TNM stage [0/I/II/III/IV], *n*	8/11/33/41/8	8/11/23/36/8	8/5/11/23/4
Depth of tumor invasion [Tis/T2/T3/T4], *n*	8/18/48/27	8/17/38/23	8/8/27/8
Lymph node metastasis [N0/N1/N2], *n*	54/26/21	44/22/20	24/14/13
Distant metastasis [M0/M1], *n*	93/8	78/8	47/4
Histological grade [G1/G2/G3/x], *n*	12/58/10/21	11/52/10/13	10/34/7/0
Primary tumor location, *n*:			
left colon	32	26	17
right colon	36	28	17
rectum	33	32	17

*n*, number of observations; F/M, female-to-male ratio; *CI*, confidence interval; TNM, tumor-node-metastasis cancer staging system.

**Table 4:** Structures of the examined oxicams.

Oxicam	Structure
Piroxicam (#**6**)	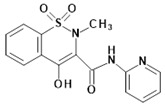
Meloxicam (#**7**)	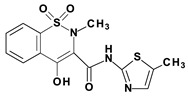
Compound #**1**	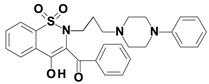
Compound #**2**	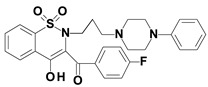
Compound #**3**	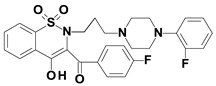
Compound #**4**	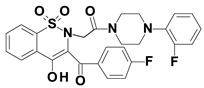
Compound #**5**	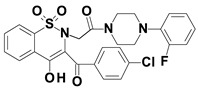

**Table 5:** Primers.

Symbol	Encoded Protein	Sequence (5′→3′)	Size [bp]
*PPIA* ^1^	Peptidylprolyl isomerase A	F: ggcaaatgctggacccaacaca R: tgctggtcttgccattcctgga	161
*RPLP0* ^1^	Ribosomal protein, large, P0	F: tcacaacaagcataccaagaagc R: gtatccgatgtccacaatgtcaag	263
*GAPDH* ^2^	Glyceraldehyde-3-phosphate dehydrogenase	F: tagattattctctgatttggtcgtattgg R: gctcctggaagatggtgatgg	223
*CCL2* ^2^	Monocyte chemotactic protein (MCP)-1	F: tctgtgcctgctgctcatag R: acttgctgctggtgattcttc	155
*CCL3* ^1^	Macrophage inflammatory protein (MIP)-1 α	F: actttgagacgagcagccagtg R: tttctggacccactcctcactg	101
*CCL4* ^1^	Macrophage inflammatory protein (MIP)-1 β	F: ggtcatacacgtactcctggac R: gcttcctcgcaactttgtggtag	140
*CCL7* ^1^	Monocyte chemotactic protein (MCP)-3	F: acagaaggaccaccagtagcca R: ggtgcttcataaagtcctggacc	117
*CCL8* ^1^	Monocyte chemotactic protein (MCP)-2	F: tatccagaggctggagagctac R: tggaatccctgacccatctctc	128
*CXCL2* ^1^	Macrophage inflammatory protein (MIP)-2	F: ggcagaaagcttgtctcaaccc R: ctccttcaggaacagccaccaa	127
*CCL19* ^1^	Macrophage inflammatory protein (MIP)-3 β	F: cgtgaggaacttccactaccttc R: gtctctggatgatgcgttctacc	131
*NAG1* ^1^	Nonsteroidal anti-inflammatory drug-activated gene (also known as growth differentiation factor (GDF)-15 and macrophage inhibitory cytokine (MIC)-1)	F: caaccagagctgggaagattcg R: cccgagagatacgcaggtgca	116
*NFKBIA* ^1^	Nuclear factor of kappa light polypeptide gene enhancer in B-cells inhibitor, alpha (IκBα)	F: tccactccatcctgaaggctac R: caaggacaccaaaagctccacg	101
*MYD88* ^1^	Myeloid differentiation factor 88 (MyD88)	F: gaggctgagaagcctttacagg R: gcagatgaaggcatcgaaacgc	129
*RELA* ^1^	Nuclear factor of kappa light polypeptide gene enhancer in B-cells, p65 subunit	F: tgaaccgaaactctggcagctg R: catcagcttgcgaaaaggagcc	135

^1^ The primer sequences were as proposed by Origene (www.origene.com (accessed on 6 February 2021)). ^2^ The primers were designed using Beacon Designer Probe/Primer Design Software (BioRad), and validated in silico (Blast analysis). The forward and reverse primer sequences are denoted by “F” and “R”, respectively. bp, base pairs.

## Data Availability

Not applicable.

## Sample Availability

Samples of the oxicam analogues are available from the authors.

## References

[1] Bray F., Ferlay J., Soerjomataram I., Siegel R.L., Torre L.A., Jemal A. (2018). Global cancer statistics 2018: GLOBOCAN estimates of incidence and mortality worldwide for 36 cancers in 185 countries. CA Cancer J. Clin..

[2] Mollica Poeta V., Massara M., Capucetti A., Bonecchi R. (2019). Chemokines and chemokine receptors: New targets for cancer immunotherapy. Front. Immunol..

[3] Korbecki J., Kojder K., Simińska D., Bohatyrewicz R., Gutowska I., Chlubek D., Baranowska-Bosiacka I. (2020). CC Chemokines in a tumor: A review of pro-cancer and anti-cancer properties of the ligands of receptors CCR1, CCR2, CCR3, and CCR4. Int. J. Mol. Sci..

[4] Korbecki J., Grochans S., Gutowska I., Barczak K., Baranowska-Bosiacka I. (2020). CC chemokines in a tumor: A review of pro-cancer and anti-cancer properties of receptors CCR5, CCR6, CCR7, CCR8, CCR9, and CCR10 ligands. Int. J. Mol. Sci..

[5] Cabrero-de Las Heras S., Martínez-Balibrea E. (2018). CXC family of chemokines as prognostic or predictive biomarkers and possible drug targets in colorectal cancer. World J. Gastroenterol..

[6] Maniewska J., Jeżewska D. (2021). Non-steroidal anti-inflammatory drugs in colorectal cancer chemoprevention. Cancers.

[7] Finetti F., Travelli C., Ercoli J., Colombo G., Buoso E., Trabalzini L. (2020). Prostaglandin E2 and cancer: Insight into tumor progression and immunity. Biology.

[8] Li Y., Soendergaard C., Bergenheim F.H., Aronoff D.M., Milne G., Riis L.B., Seidelin J.B., Jensen K.B., Nielsen O.H. (2018). COX-2-PGE2 signaling impairs intestinal epithelial regeneration and associates with TNF inhibitor responsiveness in ulcerative colitis. EBioMedicine.

[9] Roelofs H.M., Te Morsche R.H., van Heumen B.W., Nagengast F.M., Peters W.H. (2014). Over-expression of COX-2 mRNA in colorectal cancer. BMC Gastroenterol..

[10] Sheng J., Sun H., Yu F.B., Li B., Zhang Y., Zhu Y.T. (2020). The role of cyclooxygenase-2 in colorectal cancer. Int. J. Med. Sci..

[11] Saini M.K., Sanya S.N. (2014). Targeting angiogenic pathway for chemoprevention of experimental colon cancer using C-phycocyanin as cyclooxygenase-2 inhibitor. Biochem. Cell Biol..

[12] Saini M.K., Sanya S.N. (2015). Cell cycle regulation and apoptotic cell death in experimental colon carcinogenesis: Intervening with cyclooxygenase-2 inhibitors. Nutr. Cancer.

[13] Meyskens F.L., McLaren C.E., Pelot D., Fujikawa-Brooks S., Carpenter P.M., Hawk E., Kelloff G., Lawson M.J., Kidao J., McCracken J. (2008). Difluoromethylornithine plus sulindac for the prevention of sporadic colorectal adenomas: A randomized placebo-controlled, double-blind trial. Cancer Prev. Res..

[14] Burke C.A., Dekker E., Samadder N.J., Stoffel E., Cohen A. (2016). Efficacy and safety of eflornithine (CPP-1X)/sulindac combination therapy versus each as monotherapy in patients with familial adenomatous polyposis (FAP): Design and rationale of a randomized, double-blind, Phase III trial. BMC Gastroenterol..

[15] Hull M.A., Gardner S.H., Hawcroft G. (2003). Activity of the non-steroidal anti-inflammatory drug indomethacin against colorectal cancer. Cancer Treat. Rev..

[16] Zappavigna S., Cossu A.M., Grimaldi A., Bocchetti M., Ferraro G.A., Nicoletti G.F., Filosa R., Caraglia M. (2020). Anti-inflammatory drugs as anticancer agents. Int. J. Mol. Sci..

[17] Galisteo A., Jannus F., García-García A., Aheget H., Rojas S., Lupiañez J.A., Rodríguez-Diéguez A., Reyes-Zurita F.J., Quílez Del Moral J.F. (2021). Diclofenac N-derivatives as therapeutic agents with anti-inflammatory and anti-cancer effect. Int. J. Mol. Sci..

[18] Wong R.S.Y. (2019). Role of nonsteroidal anti-inflammatory drugs (NSAIDs) in cancer prevention and cancer promotion. Adv. Pharmacol. Pharm. Sci..

[19] Bjarnason I., Scarpignato C., Holmgren E., Olszewski M., Rainsford K.D., Lanas A. (2018). Mechanisms of damage to the gastrointestinal tract from nonsteroidal anti-inflammatory drugs. Gastroenterology.

[20] Xu S., Rouzer C.A., Marnett L.J. (2014). Oxicams, a class of nonsteroidal anti-inflammatory drugs and beyond. IUBMB Life.

[21] Vartiainen N., Huang C.Y., Salminen A., Goldsteins G., Chan P.H., Koistinaho J. (2001). Piroxicam and NS-398 rescue neurones from hypoxia/reoxygenation damage by a mechanism independent of cyclo-oxygenase inhibition. J. Neurochem..

[22] Szczuka I., Wierzbicki J., Serek P., Szczęśniak-Sięga B.M., Krzystek-Korpacka M. (2021). Heat shock proteins HSPA1 and HSP90AA1 are upregulated in colorectal polyps and can be targeted in cancer cells by anti-inflammatory oxicams with arylpiperazine pharmacophore and benzoyl moiety substitutions at thiazine ring. Biomolecules.

[23] Guo S., Wharton W., Moseley P., Shi H. (2007). Heat shock protein 70 regulates cellular redox status by modulating glutathione-related enzyme activities. Cell Stress Chaperones.

[24] Szczęśniak-Sięga B.M., Mogilski S., Wiglusz R.J., Janczak J., Maniewska J., Malinka W., Filipek B. (2019). Synthesis and pharmacological evaluation of novel arylpiperazine oxicams derivatives as potent analgesics without ulcerogenicity. Bioorg. Med. Chem..

[25] Krzystek-Korpacka M., Szczęśniak-Sięga B., Szczuka I., Fortuna P., Zawadzki M., Kubiak A., Mierzchała-Pasierb M., Fleszar M.G., Lewandowski Ł., Serek P. (2020). L-arginine/nitric oxide pathway is altered in colorectal cancer and can be modulated by novel derivatives from oxicam class of non-steroidal anti-inflammatory drugs. Cancers.

[26] Szczęśniak-Sięga B., Gębczak K., Gębarowski T., Maniewska J. (2018). Synthesis, COX-1/2 inhibition and antioxidant activities of new oxicam analogues designed as potential chemopreventive agents. Acta Biochim. Pol..

[27] Gurpinar E., Grizzle W.E., Piazza G.A. (2014). NSAIDs inhibit tumorigenesis, but how?. Clin. Cancer Res..

[28] Gurpinar E., Grizzle W.E., Piazza G.A. (2013). COX-independent mechanisms of cancer chemoprevention by anti-inflammatory drugs. Front. Oncol..

[29] Kaduševičius E. (2021). Novel applications of NSAIDs: Insight and future perspectives in cardiovascular, neurodegenerative, diabetes and cancer disease therapy. Int. J. Mol. Sci..

[30] Chan T.A. (2002). Nonsteroidal anti-inflammatory drugs, apoptosis, and colon-cancer chemoprevention. Lancet Oncol..

[31] Pantziarka P., Verbaanderd C., Sukhatme V., Rica Capistrano I., Crispino S., Gyawali B., Rooman I., Van Nuffel A.M., Meheus L., Sukhatme V.P. (2018). ReDO_DB: The repurposing drugs in oncology database. Ecancermedicalscience.

[32] Saini M.K., Vaiphei K., Sanyal S.N. (2012). Chemoprevention of DMH-induced rat colon carcinoma initiation by combination administration of piroxicam and C-phycocyanin. Mol. Cell Biochem..

[33] Calaluce R., Earnest D.L., Heddens D., Einspahr J.G., Roe D., Bogert C.L., Marshall J.R., Alberts D.S. (2000). Effects of piroxicam on prostaglandin E2 levels in rectal mucosa of adenomatous polyp patients: A randomized phase IIb trial. Cancer Epidemiol. Biomark. Prev..

[34] Geller M.A., Bui-Nguyen T.M., Rogers L.M., Ramakrishnan S. (2010). Chemotherapy induces macrophage chemoattractant protein-1 production in ovarian cancer. Int. J. Gynecol. Cancer.

[35] Hatnapure G.D., Keche A.P., Rodge A.H., Birajdar S.S., Tale R.H., Kamble V.M. (2012). Synthesis and biological evaluation of novel piperazine derivatives of flavone as potent anti-inflammatory and antimicrobial agent. Bioorg. Med. Chem. Lett..

[36] Dogruer D., Kupeli E., Yesilada E., Sahin M.F. (2004). Synthesis of new 2-[1(2H)-Phthalazinon-2-yl]-acetamide and 3-[1(2H)-Phthalazinon-2-yl]-propanamide derivatives as antinociceptive and anti-inflammatory agents. Arch. Pharm. Med. Chem..

[37] Bravatà V., Minafra L., Forte G.I., Cammarata F.P., Russo G., Di Maggio F.M., Augello G., Lio D., Gilardi M.C. (2017). Cytokine profile of breast cell lines after different radiation doses. Int. J. Radiat. Biol..

[38] Tang S., Yuan X., Song J., Chen Y., Tan X., Li Q. (2019). Association analyses of the JAK/STAT signaling pathway with the progression and prognosis of colon cancer. Oncol. Lett..

[39] Bieche I., Chavey C., Andrieu C., Busson M., Vacher S., Le C.L., Guinebretiere J.M., Burlinchon S., Lidereau R., Lazennec G. (2007). CXC chemokines located in the 4q21 region are up-regulated in breast cancer. Endocr. Relat. Cancer.

[40] Gupta V., Yeo G., Kawakubo H., Rangnekar V., Ramaswamy P., Hayashida T., MacLaughlin D.T., Donahoe P.K., Maheswaran S. (2007). Mullerian-inhibiting substance induces Gro-beta expression in breast cancer cells through a nuclear factor-kappaBdependent and Smad1-dependent mechanism. Cancer Res..

[41] Cao Y., Chen C., Weatherbee J.A., Tsang M., Folkman J. (1995). Grobeta, a -C-X-C- chemokine, is an angiogenesis inhibitor that suppresses the growth of Lewis lung carcinoma in mice. J. Exp. Med..

[42] Matsuo N., Azuma K., Sasada T. (2019). Assessment of soluble immune mediators as potential biomarkers during immune checkpoint inhibitor therapy. Oncotarget.

[43] Cuenca R.E., Azizkhan R.G., Haskill S. (1992). Characterization of GRO alpha, beta and gamma expression in human colonic tumours: Potential significance of cytokine involvement. Surg. Oncol..

[44] McLean M.H., Murray G.I., Stewart K.N., Norrie G., Mayer C., Hold G.L., Thomson J., Fyfe N., Hope M., Mowat N.A. (2011). The inflammatory microenvironment in colorectal neoplasia. PLoS ONE.

[45] Doll D., Keller L., Maak M., Boulesteix A.L., Siewert J.R., Holzmann B., Janssen K.P. (2010). Differential expression of the chemokines GRO-2, GRO-3, and interleukin-8 in colon cancer and their impact on metastatic disease and survival. Int. J. Colorectal Dis..

[46] Baek S.J., Wilson L.C., Lee C.H., Eling T.E. (2002). Dual function of nonsteroidal anti-inflammatory drugs (NSAIDs): Inhibition of cyclooxygenase and induction of NSAID-activated gene. J. Pharmacol. Exp. Ther..

[47] Moon Y. (2017). NSAID-activated gene 1 and its implications for mucosal integrity and intervention beyond NSAIDs. Pharmacol. Res..

[48] Eling T.E., Baek S.J., Shim M., Lee C.H. (2006). NSAID activated gene (NAG-1), a modulator of tumorigenesis. J. Biochem. Mol. Biol..

[49] Yoshioka H., Kamitani H., Watanabe T., Eling T.E. (2008). Nonsteroidal anti-inflammatory drug-activated gene (NAG-1/GDF15) expression is increased by the histone deacetylase inhibitor trichostatin A. J. Biol. Chem..

[50] Choi H.J., Do K.H., Park J.H., Kim J., Yu M., Park S.H., Moon Y. (2016). Early epithelial restitution by nonsteroidal anti-inflammatory drug-activated gene 1 counteracts intestinal ulcerative injuries. J. Immunol..

[51] Hong Q., Li B., Cai X., Lv Z., Cai S., Zhong Y., Wen B. (2021). Transcriptomic analyses of the adenoma-carcinoma sequence identify hallmarks associated with the onset of colorectal cancer. Front. Oncol..

[52] Yang C., Chen D., Huang K., Zhang H., Xu D., Tian Y., Zhang J. (2006). The expression of chemokine MCP-1 in colorectal carcinoma and its relationship to the infiltration of macrophage. Chin. Ger. J. Clin. Oncol..

[53] Bailey C., Negus R., Morris A., Ziprin P., Goldin R., Allavena P., Peck D., Darzi A. (2007). Chemokine expression is associated with the accumulation of tumour associated macrophages (TAMs) and progression in human colorectal cancer. Clin. Exp. Metastasis.

[54] Yoshidome H., Kohno H., Shida T., Kimura F., Shimizu H., Ohtsuka M., Nakatani Y., Miyazaki M. (2009). Significance of monocyte chemoattractant protein-1 in angiogenesis and survival in colorectal liver metastases. Int. J. Oncol..

[55] Wolf M.J., Hoos A., Bauer J., Boettcher S., Knust M., Weber A., Simonavicius N., Schneider C., Lang M., Sturzl M. (2012). Endothelial CCR2 signaling induced by colon carcinoma cells enables extravasation via the JAK2-Stat5 and p38MAPK pathway. Cancer Cell.

[56] Chu S., Wang H., Yu M. (2017). A putative molecular network associated with colon cancer metastasis constructed from microarray data. World J. Surg. Oncol..

[57] Xu M., Wang S., Qi Y., Chen L., Frank J.A., Yang X.H., Zhang Z., Shi X., Luo J. (2016). Role of MCP-1 in alcohol-induced aggressiveness of colorectal cancer cells. Mol. Carcinog..

[58] Diakowska D., Krzystek-Korpacka M. (2020). Local and systemic interleukin-32 in esophageal, gastric, and colorectal cancers: Clinical and diagnostic significance. Diagnostics.

[59] Bednarz-Misa I., Fortuna P., Fleszar M.G., Lewandowski Ł., Diakowska D., Rosińczuk J., Krzystek-Korpacka M. (2020). Esophageal squamous cell carcinoma is accompanied by local and systemic changes in L-arginine/NO pathway. Int. J. Mol. Sci..

[60] Lewandowska P., Wierzbicki J., Zawadzki M., Agrawal A., Krzystek-Korpacka M. (2020). Biphasic expression of atypical chemokine receptor (ACKR) 2 and ACKR4 in colorectal neoplasms in association with histopathological findings. Biomolecules.

[61] Patel A., Tripathi G., Gopalakrishnan K., Williams N., Arasaradnam R.P. (2015). Field cancerisation in colorectal cancer: A new frontier or pastures past?. World J. Gastroenterol..

[62] Lee J.M., Han Y.D., Cho M.S., Hur H., Min B.S., Lee K.Y., Kim N.K. (2019). Impact of tumor sidedness on survival and recurrence patterns in colon cancer patients. Ann. Surg. Treat. Res..

[63] Farmaki E., Chatzistamou I., Kaza V., Kiaris H. (2016). A CCL8 gradient drives breast cancer cell dissemination. Oncogene.

[64] McClellan J.L., Davis J.M., Steiner J.L., Enos R.T., Jung S.H., Carson J.A., Pena M.M., Carnevale K.A., Berger F.G., Murphy E.A. (2012). Linking tumor-associated macrophages, inflammation, and intestinal tumorigenesis: Role of MCP-1. Am. J. Physiol. Gastrointest. Liver Physiol..

[65] Song M., Sasazuki S., Camargo M.C., Shimazu T., Charvat H., Yamaji T., Sawada N., Kemp T.J., Pfeiffer R.M., Hildesheim A. (2018). Circulating inflammatory markers and colorectal cancer risk: A prospective case-cohort study in Japan. Int. J. Cancer.

[66] Popivanova B.K., Kostadinova F.I., Furuichi K., Shamekh M.M., Kondo T., Wada T., Egashira K., Mukaida N. (2009). Blockade of a chemokine, CCL2, reduces chronic colitis-associated carcinogenesis in mice. Cancer Res..

[67] Lu J., Zhao J., Feng H., Wang P., Zhang Z., Zong Y., Ma J., Zheng M., Lu A. (2014). Antitumor efficacy of CC motif chemokine ligand 19 in colorectal cancer. Dig. Dis. Sci..

[68] Xu Z., Zhu C., Chen C., Zong Y., Feng H., Liu D., Feng W., Zhao J., Lu A. (2018). CCL19 suppresses angiogenesis through promoting miR-206 and inhibiting Met/ERK/Elk-1/HIF-1α/VEGF-A pathway in colorectal cancer. Cell Death Dis..

[69] Chen L., Lu D., Sun K., Xu Y., Hu P., Li X., Xu F. (2019). Identification of biomarkers associated with diagnosis and prognosis of colorectal cancer patients based on integrated bioinformatics analysis. Gene.

[70] Liu X., Wang B., Li Y., Hu Y., Li X., Yu T., Ju Y., Sun T., Gao X., Wei Y. (2019). Powerful anticolon tumor effect of targeted gene immunotherapy using folate-modified nanoparticle delivery of CCL19 to activate the immune system. ACS Cent. Sci..

[71] Lu J., Ma J., Cai W., Wangpu X., Feng H., Zhao J., Guan S., Zong Y., Lu A. (2015). CC motif chemokine ligand 19 suppressed colorectal cancer in vivo accompanied by an increase in IL-12 and IFN-γ. Biomed. Pharmacother..

[72] De la Fuente López M., Landskron G., Parada D., Dubois-Camacho K., Simian D., Martinez M., Romero D., Roa J.C., Chahuán I., Gutiérrez R. (2018). The relationship between chemokines CCL2, CCL3, and CCL4 with the tumor microenvironment and tumor-associated macrophage markers in colorectal cancer. Tumor Biol..

[73] Krzystek-Korpacka M., Diakowska D., Kapturkiewicz B., Bębenek M., Gamian A. (2013). Profiles of circulating inflammatory cytokines in colorectal cancer (CRC), high cancer risk conditions, and health are distinct. Possible implications for CRC screening and surveillance. Cancer Lett..

[74] Nishikawa G., Kawada K., Nakagawa J., Toda K., Ogawa R., Inamoto S., Mizuno R., Itatani Y., Sakai Y. (2019). Bone marrow-derived mesenchymal stem cells promote colorectal cancer progression via CCR5. Cell Death Dis..

[75] Liao Y.Y., Tsai H.C., Chou P.Y., Wang S.W., Chen H.T., Lin Y.M., Chiang I.P., Chang T.M., Hsu S.K., Chou M.C. (2016). CCL3 promotes angiogenesis by dysregulation of miR-374b/VEGF-A axis in human osteosarcoma cells. Oncotarget.

[76] Pender S.L., Chance V., Whiting C.V., Buckley M., Edwards M., Pettipher R., MacDonald T.T. (2005). Systemic administration of the chemokine macrophage inflammatory protein 1alpha exacerbates inflammatory bowel disease in a mouse model. Gut.

[77] Sasaki S., Baba T., Shinagawa K., Matsushima K., Mukaida N. (2014). Crucial involvement of the CCL3-CCR5 axis-mediated fibroblast accumulation in colitis-associated carcinogenesis in mice. Int. J. Cancer.

[78] Väyrynen J.P., Kantola T., Väyrynen S.A., Klintrup K., Bloigu R., Karhu T., Mäkelä J., Herzig K.H., Karttunen T.J., Tuomisto A. (2016). The relationships between serum cytokine levels and tumor infiltrating immune cells and their clinical significance in colorectal cancer. Int. J. Cancer.

[79] Slattery M.L., Mullany L.E., Sakoda L., Samowitz W.S., Wolff R.K., Stevens J.R., Herrick J.S. (2018). The NF-κB signalling pathway in colorectal cancer: Associations between dysregulated gene and miRNA expression. J. Cancer Res. Clin. Oncol..

[80] Allen F., Bobanga I.D., Rauhe P., Barkauskas D., Teich N., Tong C., Myers J., Huang A.Y. (2017). CCL3 augments tumor rejection and enhances CD8+ T cell infiltration through NK and CD103+ dendritic cell recruitment via IFNγ. Oncoimmunology.

[81] Luo X., Yu Y., Liang A., Xie Y., Liu S., Guo J., Wang W., Qi R., An H., Zhang M. (2004). Intratumoral expression of MIP-1beta induces antitumor responses in a pre-established tumor model through chemoattracting T cells and NK cells. Cell Mol. Immunol..

[82] Krzyżak E., Szczęśniak-Sięga B., Malinka W. (2014). Synthesis and thermal behaviour of new benzo-1,2-thiazine long-chain aryl-piperazine derivatives. J. Therm. Anal. Calorim..

[83] Szczęśniak-Sięga B., Maniewska J., Poła A., Środa-Pomianek K., Malinka W., Michalak K. (2014). Synthesis of new Piroxicam derivatives and their influence on lipid bilayers. Acta Pol. Pharm. Drug Res..

[84] Maniewska J., Szczęśniak-Sięga B., Poła A., Środa-Pomianek K., Malinka W., Michalak K. (2014). The interaction of new piroxicam analogues with lipid bilayers—A calorimetric and fluorescence spectroscopic study. Acta Pol. Pharm. Drug Res..

[85] Hellemans J., Vandesompele J., Kennedy S., Oswald N. (2011). qPCR data analysis—Unlocking the secret to successful results. PCR Troubleshooting and Optimization: The Essential Guide.

[86] Krzystek-Korpacka M., Diakowska D., Bania J., Gamian A. (2014). Expression stability of common housekeeping genes is differently affected by bowel inflammation and cancer: Implications for finding suitable normalizers for inflammatory bowel disease studies. Inflamm. Bowel Dis..

[87] Krzystek-Korpacka M., Hotowy K., Czapinska E., Podkowik M., Bania J., Gamian A., Bednarz-Misa I. (2016). Serum availability affects expression of common house-keeping genes in colon adenocarcinoma cell lines: Implications for quantitative real-time PCR studies. Cytotechnology.

[88] Krzystek-Korpacka M., Zawadzki M., Lewandowska P., Szufnarowski K., Bednarz-Misa I., Jacyna K., Witkiewicz W., Gamian A. (2019). Distinct chemokine dynamics in early postoperative period after open and robotic colorectal surgery. J. Clin. Med..

[89] Krzystek-Korpacka M., Zawadzki M., Kapturkiewicz B., Lewandowska P., Bednarz-Misa I., Gorska S., Witkiewicz W., Gamian A. (2018). Subsite heterogeneity in the profiles of circulating cytokines in colorectal cancer. Cytokine.

